# Exploring the synthesis, characterization, and corrosion inhibition of new tris-thiosemicarbazone derivatives for acidic steel settings using computational and experimental studies

**DOI:** 10.1038/s41598-024-64199-x

**Published:** 2024-06-10

**Authors:** Ahmed M. Abuelela, Mahmoud A. Bedair, Ehab S. Gad, Y. F. El-Aryan, Wael Abdelgayed Ahmed Arafa, Asmaa K. Mourad, H. Nady, Salah Eid

**Affiliations:** 1 Department of Chemistry, College of Science, King Faisal University, 31982 Al-Hassa, Saudi Arabia; 2 Department of Chemistry, College of Science, University of Bisha, P.O. Box 511, 61922 Bisha, Saudi Arabia; 3 Chemistry Department, College of Science, Jouf University, P. O. Box 2014, Sakaka, Jouf Saudi Arabia; 4 Chemistry Department, Faculty of Science, Fayoum University, P. O. Box 63514, Fayoum, Egypt; 5 Chemistry Department, Faculty of Science, Benha University, Benha, Egypt

**Keywords:** Mild steel, Tri-thiocarboxyhydrazones, Acid corrosion, Thermotical studies, Electrochemistry, Physical chemistry, Theoretical chemistry

## Abstract

A novel two tri-thiosemicarbazones derivatives, namely 2,2',2''-((2-Hydroxybenzene-1,3,5-triyl)tris(methanylylidene))tris(*N*-benzylhydrazine-1-carbothioamide) (**HBC**) and 2,2',2''-((2-hydroxybenzene-1,3,5-triyl) tris (methanylylidene)) tris (*N*-allylhydrazine-1-carbothioamide) (**HAC**), have been synthesized and their chemical structures were determined using different spectroscopic and analytical approaches. Then, utilizing methods including open circuit potential, potentiodynamic polarization, and electrochemical impedance spectroscopy, the inhibitory effect of the synthesized thiosemicarbazones on mild steel (MS) in an acidic environment (0.5 M H_2_SO_4_) was thoroughly investigated. Remarkably, raising the concentration of our recently synthesized tri-thiosemicarbazones HBC and HAC increased the inhibitory efficiency values. The *η* values of the two investigated tri-thiosemicarbazones derivatives (HAC and HBC), at each concentration are extremely high, and the maximum values of the efficiencies are 98.5% with HAC and 98.8% with HBC at the 800 ppm. The inhibitors adsorbed on the mild steel surface and generated a charge and mass movement barrier that protected the metal from hostile ions. According to polarization curves, *HBC* and *HAC* act as mixed-type inhibitors. Electrochemical impedance testing revealed a notable rise in charge transfer resistance (R_ct_) readings to 4930-Ω cm^2^, alongside a reduction in the Constant Phase Element (CPE) value to 5.81 μF, suggesting increased effectiveness in preventing corrosion. Also, density functional theory (DFT) was applied to investigate the assembled tri-thiosemicarbazones HBC and HAC. Moreover, the adsorption mechanism of *HBC* and *HAC* on the mild steel surface was explored using Monte Carlo simulation. Finally, the theoretical outputs were discovered to support the practical outcomes.

## Introduction

One of the most important elements that man has employed from the beginning of time is iron. Despite its susceptibility for corrosion, mild steel is one of the most significant substances ever created and has been used in a wide range of industries and machines. Mild steel is utilized to create structural forms and sheets, used automobiles, furniture, pipelines, bridges, decorations, and wire^1,2^. Certainly, the utilization of crude oil and natural gas stands as one of the most crucial energy sources, involving a cycle from extraction in wells to the refining process. Subsequently, transportation occurs through pipelines constructed from steel, as well as storage in tanks and the infrastructure of refining facilities^3^. The investigation of the corrosion behavior of steel in different aqueous media have been received a great attention. Acidic environments, which are typically present in industrial and chemical processes, cause M-Steel to corrode quickly^4–6^. Acid solutions, especially H_2_SO_4_ and HCl are widely used in different industrial applications, such as petroleum industry, pickling of iron, chemical cleaning and descaling of boilers. Inhibitors are a frequent and effective way to protect metals in corrosive conditions^7–9^.

It is well recognized that organic compounds, particularly those with heteroatoms like nitrogen, sulfur, and oxygen, are efficient corrosion inhibitors^10^. The effectiveness of these chemical inhibitors is related to their capacity to bind to the metallic surface^11,12^. Recent research has focused on thiosemicarbazones, which have been found to provide good inhibition because of structure contains heteroatoms with free electron pairs in addition to a conjugated double bond that can promote adsorption on M-Steel^13–15^. Thiosemicarbazones have been a target of research in recent years owing to their potential utilization in chemistry and related industries^13,16,17^. Compounds comprising such motifs emerged as a prominent class of N, S-donor ligands over time because of diverse donor behaviors, molecular variety, and biological uses. Such compounds perform as proper ligands since they have superior coordination capability, selectivity, and stability when utilized with a broad range of metal ions^13,17–19^. Further, they can coordinate with several metal ions as keto form (neutral) or enol form (anionic) ligands, and they could adopt a range of distinct coordination patterns. In some cases, the formation of a single complex possessing both deprotonated and neutral thiosemicarbazone species is conceivable^20^. The chelating efficiency of the thiosemicarbazone moiety could be improved by introducing appropriate donor atoms in its framework, thereby making the ligand polydentate^21^. Furthermore, thiosemicarbazones and their metal were exhibited promises in the therapy of a variety of ailments^13,16,18,19^. Additionally, thiosemicarbazones and their metal complexes have a considerable spectrum of biological and therapeutic applications, particularly, antimalarial, antiviral, antifungal, and anticancer activities^22,23^. Thiosemicarbazones derivatives are widely applied as inhibitors for steel in sulfuric acid environment. Zhang et al. synthesized and investigated the corrosion inhibitory effects of various benzaldehyde thiosemicarbazone derivatives (BST, PBST, and OCT) on the corrosion of mild steel in a 0.5 M H_2_SO_4_ solution^24^. They found a significant positive relationship between concentration and inhibition efficiency. Qusay A. Jawad and collaborators explored how a new thiosemicarbazone, specifically 2-(2,4-di methoxy benzylidene) hydrazinecarbothioamide (DMBHC), affects the corrosion inhibition of mild steel in a sulfuric acid environment. The concentration of 0.5 × 10^–3^ M demonstrated the highest inhibitory efficiency, reaching 94.3%^25^. Furthermore, Honghong Zhang and colleagues synthesized novel halogen-substituted benzaldehyde thiosemicarbazone derivatives and assessed their efficacy as corrosion inhibitors for mild steel in 1 M HCl. The research demonstrated a direct relationship between concentration and inhibitory effectiveness, reaching a peak of 94.3% at a concentration of 400 × 10^–6^ M^26^. Some publications primarily focus on electrochemical assessments, offering only a superficial examination of structural elements. This study aims to provide a comprehensive overview of structural constituents and establish connections with electrochemical performance within a single publication. To conduct an integrated structural analysis, we applied FMO and NBO methodologies to investigate corrosion effectiveness at the molecular level. Our utilization of NBO analysis allowed us to prioritize functional groups and specific molecular sites for facilitating electron transfer with the metal surface. The purpose of this study is to assess the inhibitory effect of some novel tris-thiosemicarbazones compounds on mild steel (MS) corrosion in 0.5 M H_2_SO_4_. Open circuit potential, potentiodynamic polarization, and electrochemical impedance spectroscopy (EIS) measurements were used in this study. Theoretical calculations were also used to validate the experimental results.

## Materials and methods

### Materials

The chemical composition of the relevant MS was (weight%). Fe = 99.77, C = 0.06495 which were chosen for the present study and exposed to the hostile medium. The MS working electrode was polished to a mirror shine using emery sheets of varying grits (200–2000), cleaned, and washed with bi-distilled water and acetone to get rid of grease. All of the chemicals used to generate the test solutions were of Sigma-Aldrich Co., and all experiments were performed at 30 °C.

### Synthesis of inhibitors derivatives

**Figure 1:**
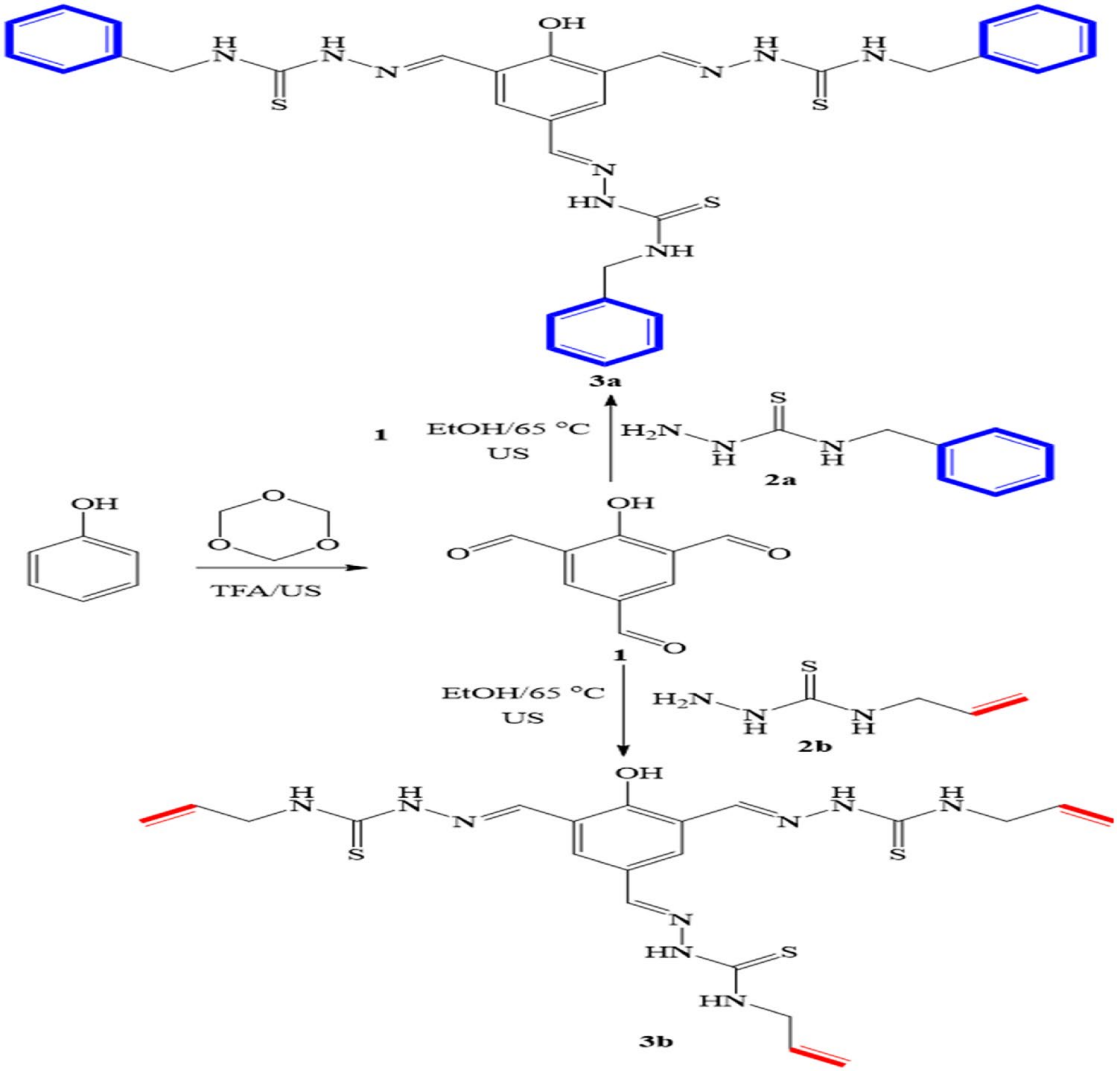
An ultrasound-assisted protocol for the synthesis of the (HAC and HBC).

A solution of 2-hydroxybenzene-1,3,5-tricarbaldehyde (**1**, 3.0 mmol, 0.534 g) and an appropriate *N*-substituted hydrazine carbothioamides (**2a,b**, 3.0 mmol) in EtOH (15.0 mL) was sonicated at 65°C for 25 min (TLC). The isolated solid was filtered off, washed with EtOH (3 × 3.0 mL), dried under vacuo, and crystallized from dioxane/DMF. The molecular structure of **3a** and **3b** are given in Fig. 1.

#### 2,2',2''-((2-Hydroxybenzene-1,3,5-triyl) tris (methanylylidene)) tris (N-benzylhydrazine-1-carbothioamide) (3a, HBC)

**Figure 2:**
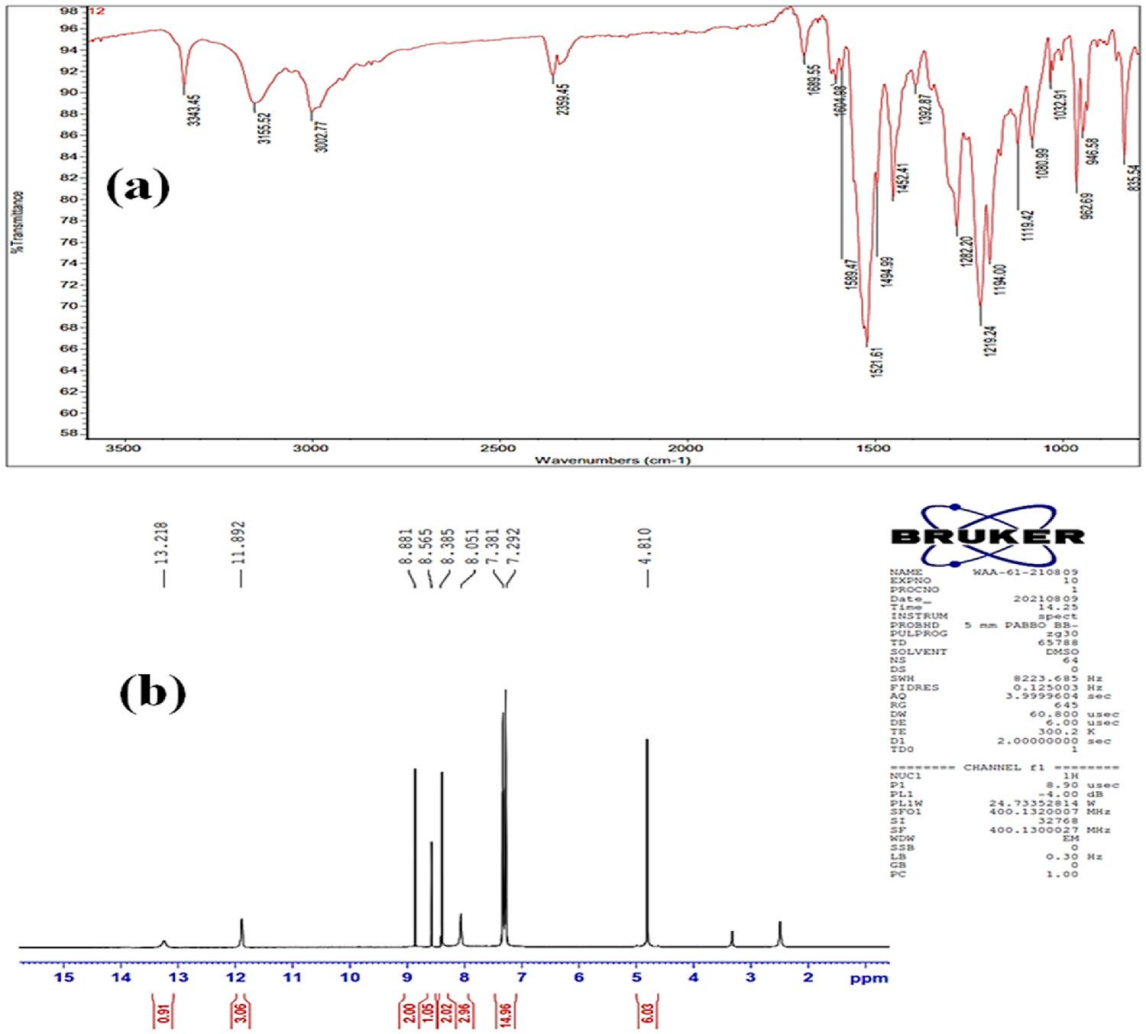
The FTIR spectrum (**a**), and ^1^H NMR spectrum spectra of **HBC** compound.

Yellow crystals; 98%; mp322-325°C. IR (KBr) (Fig. 2a): 3343, 3155 (broad bands, NH&OH), 1589 (C=N&C=C), 1452 (C=S). ^1^H NMR (400 MHz, DMSO-*d6*) (Fig. 2b): *δ* = 13.21 (br, 1H, O*H*), 11.89 (br, 3H, N*H*-N=CH), 8.88 (s, 2H, C*H*=N), 8.56 (s, 1H, C*H*=N), 8.38 (s, 2H, Ar–H), 8.05 (br, 3H, N*H*-CH_2_), 7.38–7.29 (m, 15H, Ar–H), 4.81 ppm (s, 6H, C*H*_2_). HRMS*m/z*: [M]^+^ calcd for C_33_H_33_N_9_OS_3_:667.1973, found:667.1972. Anal. Calcd for C_33_H_33_N_9_OS_3_: C, 59.35; H, 4.98; N, 18.88%. Found: C, 59.31; H, 4.99; N, 18.80%.

#### 2,2',2''-((2-Hydroxybenzene-1,3,5-triyl) tris (methanylylidene)) tris (***N***-allylhydrazine-1-carbothioamide) (3b, HAC)

**Figure 3:**
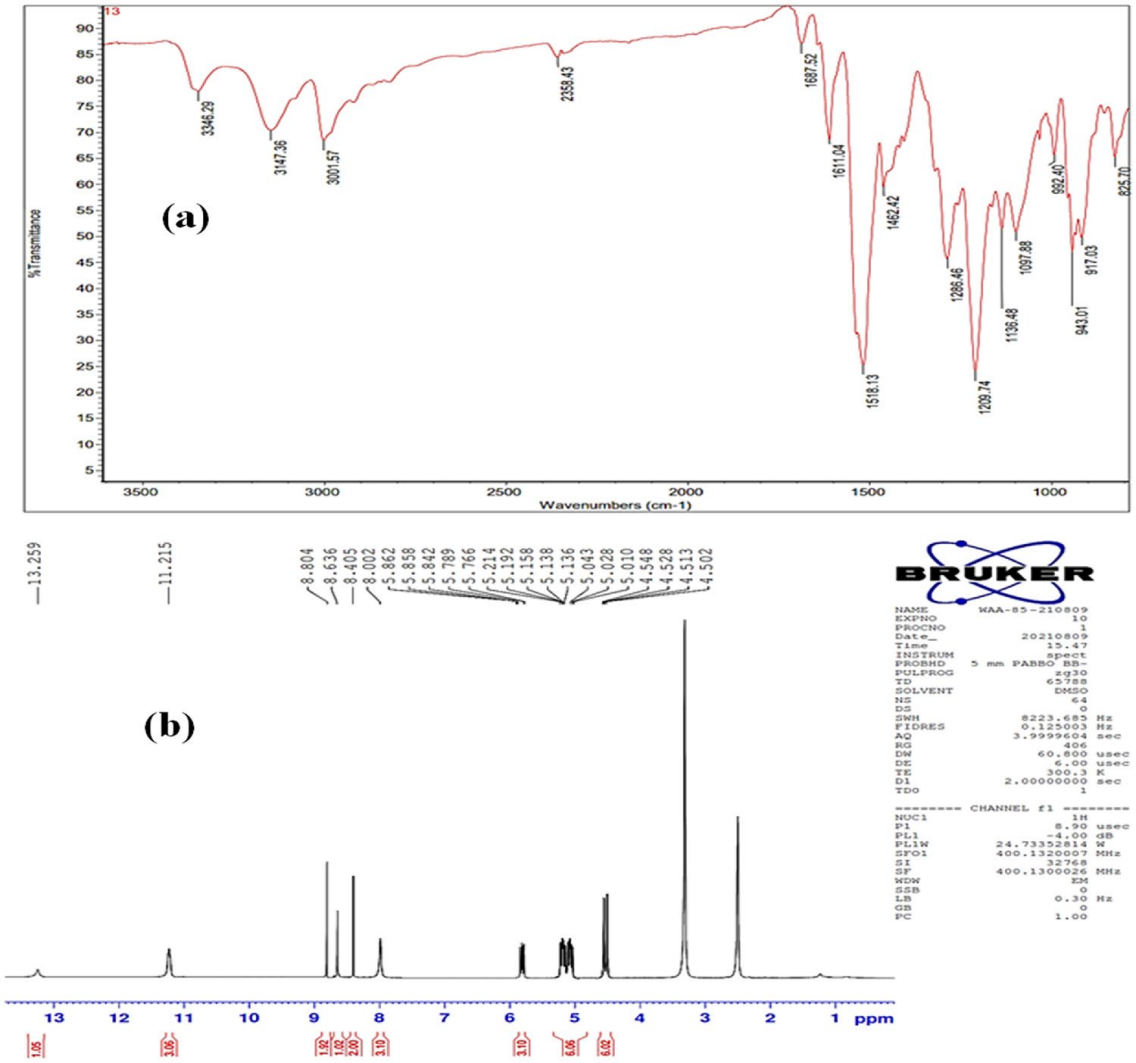
The FTIR spectrum (**a**), and ^1^H NMR spectrum spectra of **HAC** compound.

Yellow crystals; yield 96%; mp 303–304 °C. IR (KBr) (Fig. 3a): 3346, 3147 (broad bands, NH&OH), 1611 (C=N & C=C), 1462 (C=S). ^1^H NMR (400 MHz, DMSO-*d6*) (Fig. 3b): *δ* = 13.25 (s, 1H, OH), 11.21 (br,3H, N*H*-N=CH), 8.80 (s, 2H, C*H*=N), 8.63 (s, 1H, C*H*=N), 8.40 (s, 2H, Ar–H), 8.00 (br, 3H, N*H*-CH_2_), 5.86–5.76 (m, 3H, CH_2_=C*H*-CH_2_), 5.21–5.01 (m, 6H, C*H*_2_=CH-CH_2_), 4.54–4.50 ppm (m, 6H, CH_2_=CH-C*H*_2_). HRMS*m/z*: [M]^+^ calcd for C_21_H_27_N_9_OS_3_:517.1505, found:517.102. Anal. Calcd for C_21_H_27_N_9_OS_3_: C, 48.72; H, 5.26; N, 24.35%. Found: C, 48.85; H, 5.19; N, 24.31%.

### Electrochemical measurements

All electrochemical measurements were achieved with *Versa STAT* 4 and the *Versa Studio* electrochemistry software suite. The working electrode was rubbed with emery sheets of various grades up to 2000 grit. As counter and reference electrodes, platinum wire and saturated calomel electrodes were used. Before beginning any experiment, the steel electrode was allowed to reach steady-state potential. The scan rate for the potentiodynamic polarization technique is 5 mV/sec. In electrochemical impedance spectroscopy (EIS) studies, the frequency ranges 100 kHz to 100 MHz are used.

### Computational details

The geometries of the HAC and HBC molecules were constructed using the sketching tools in Gaussview 6 software^27^. Their structural parameters (bond length, bond angle and dihedral angle) in the gas and aqueous forms were optimized to the minimum energy using Gaussian 09 software package^28^. The optimization methods used the Lee–Yang–Parr correlation functional and Becke-style 3-Parameter density Functional Theory (DFT-B3LYP) at 6–31G(d,p) basis set, which had a tolerable processing cost, less time and high accuracy^29,30^. The optimization processes were also done using DMol^3^ module implemented in Materials studio software^31^ at local spin density approximation using the Perdew–Wang correlation function (LDA and PWC), (DND 4.4) Basis Set and COSMO control for water as solvent^32^. The occurrence of HAC and HBC molecules on steel surface was simulated by Molecular dynamics (MD) and Monte Carlo (MC) simulations using Forcite dynamic and adsorption locator modules in absence and presence of the corrosive environment (200 H_2_O/20 H3O^+^/10 SO_4_^--^). For this purpose, Fe (110) slab with a dimension of 24.82Å × 24.82Å × 48.24 Å was used to simulate steel. Molecular dynamics (MD) was done by using Nose thermostat where the temperature was set at 298 K. A NVT ensemble with simulation time of 50 ps is chosen where the time step of 1 fs. The energy was performed using COMPASS II as the force field.

### Institutional review board statement

All procedures were performed in accordance with the Guidelines for Care and Use of Laboratory of Benha University and approved by the Ethics Committee of faculty of science (BUFS-REC-2024-132 chm).

## Results and discussion

### Synthesis and characterization

We began our investigation by synthesizing 2-hydroxybenzene-1,3,5-tricarbaldehyde (**1**) employing the published method^33^ with some modifications. The sonication of the commercially available phenol (1.0 equiv) and 1,3,5-trioxane (5.0 equiv) in trifluoroacetic acid (TFA) was demonstrated to be capable of introducing three aldehydic groups into the phenol molecule in a one-step procedure. In comparison with the published methods^33^, the present protocol afforded the desired tricarbaldehyde (**1**) quantitatively in a short reaction period. Subsequently, the trialdehyde derivative (**1**) has been then subject to condensation reactions with thiosemicarbazde derivatives (**2a,b**) under different conditions. After several trials, the optimum conditions for this condensation reaction were specified as follows: a mixture of 3.0 mmol of tricarbaldehyde (**1**) and 3.0 mmol of thiosemicarbazides (**2a,b**) in 20.0 mL EtOH was sonicated (80%) at 65 °C for 30 min. The targeted derivatives (**3a,b**) were obtained in quantitative yields (95 and 98%, Fig. 1). The proposed structures of the targeted derivatives were confirmed via several spectroscopic methods (NMR, and FT-IR,) (cf. Figs. 2, 3). For example, Fourier transform infrared (FT-IR) spectra of the four condensed derivatives approved the disappearance of distinctive signals belonging to the aldehydic carbonyl groups. The broadness of OH bands between 3346 cm and 3001 cm^−1^indicates the stretching mode of hydroxyl and NH groups. Furthermore, the typical sharp band at 1611 cm^−1^ is assigned to the stretching vibration of the allylic double bond of derivative **3b**. Further, the bands recorded around 1500 cm^−1^ is due to the vibrational mode of the aromatic C=C bonds and the newly formed C=N bonds. Moreover, the ^1^H NMR of these derivatives (**3a,b**) revealed characteristic singlets around *δ* 8.7 ppm attributed to the methine proton. Interestingly, these relatively high values of methine protons indicating the stereoselectivity of the present reaction; the thermodynamically stable *E*-isomers were obtained while the less stable *Z-*isomers were not detected^34^. The mass spectra of the designed compounds displayed molecular ion peaks at the appropriate *m/z* values.

### Electrochemical measurements

#### Open circuit potential (OCP)

**Figure 4:**
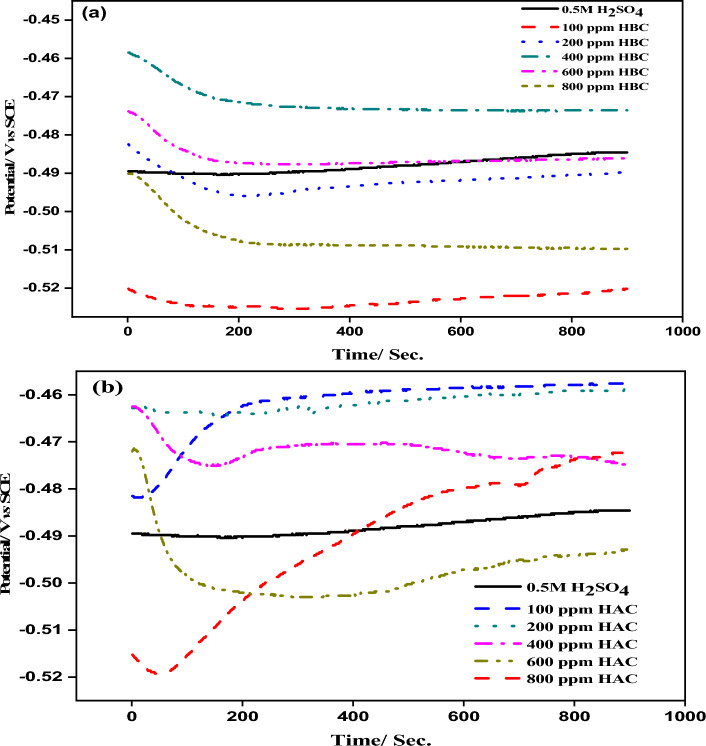
Open-circuit potential of mild steel in 0.5 M H_2_SO_4_ in absence and presence of different concentration of HBC **(a)** and HAC (**b**).

In the absence and presence of various concentrations of tri-thiosemicarbazone derivatives (HBC and HAC), the open circuit potential *vs*. time curves for MS in 0.5 M H_2_SO_4_ are shown in Fig. 4. A thorough examination of OCP vs. time curves indicates that the steady-state potential shifts negatively in the presence of HBC and HAC. As the concentration of **HBC** and **HAC** increases, the OCP becomes more negative. The contradicting variations of the curves in proportion to uninhibited MS revealed that the cathodic reaction was dominant. The properties of the curled sample are markedly different from those of the unrestricted sample. For MS electrode, the OCP curves were practically straight, indicating that steady-state potential had been reached^35,36^.

#### Potentiodynamic polarization (PDP)

**Figure 5:**
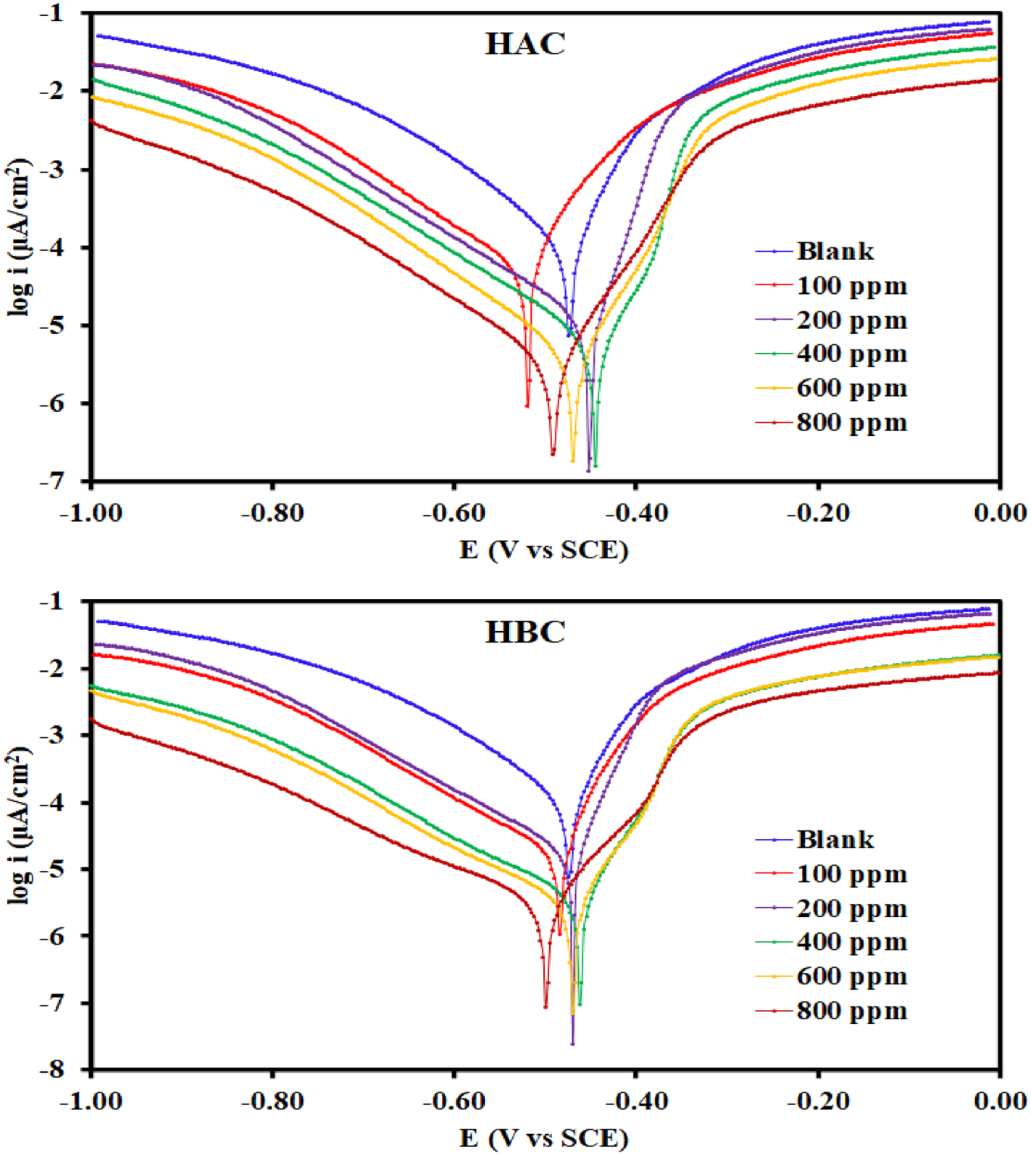
Potentiodynamic polarization curves for the corrosion of Steel in 0.5 M H_2_SO_4_ in absence and presence of different concentrations of HAC and HBC compound at 303 K.

The potentiodynamic polarization (PDP) curves of MS were carried out after holding the alloys at the open-circuit potential in stagnant naturally aerated 0.5 M H_2_SO_4_ in inhibitors free and inhibitors containing solutions (100–800 ppm). Figure 5 presents the results of the effect of tri-thiosemicarbazones derivatives concentrations on cathodic and anodic polarization curves of MS in 0.5 M H_2_SO_4_ at 303 K. The corrosion current density, *i*_*corr*_, corrosion potential, *E*_*corr*_, surface coverage, *θ*, inhibition efficiency, *η*, anodic Tafel slopes, *β*a, and cathodic Tafel slopes, *β*_c_, were calculated using PDP data and presented in Table 1. The curves showed that the synthesized tri-thiosemicarbazone derivatives (HBC and HAC) are very effective inhibitors for the MS corrosion. When the effect of the two inhibitors compared to each other at 800 ppm, it was noticed that HBC is more effective on the MS surface than HAC inhibitor (Fig. 5). The polarization curves shift directly to lower current densities as HBC and HAC concentrations are increased, dramatically slowing the rate at which MS corrodes. The fact that each curve in Fig. 5 is parallel to the others shows that the addition of the HBC and HAC has no effect on the mechanism of reactions, and that the corrosion processes can be controlled simply by limiting the rates of reactions^37,38^. The values displayed in Table 1 indicated that *i*_corr_ decreases sharply from 2326 μA cm^−2^ (for the uninhibited tested media) to 34.39 μA cm^−2^ (with 800 ppm of HAC) and 28.26 μA cm^−2^ (with 800 ppm of HBC**)**. The values also suggest that HAC and HBC adsorb on the metal surface, which suppresses the dissolution of metals as well as reduction processes^39^. Additionally, it has been noted that the addition of HAC and HBC to the corrosive solution has altered the *E*_*corr*_ values of the non-inhibitor systems. At 100 ppm, the attained efficiency ranged from 63.16% (HAC) and 75.86% (HBC) to its maximum value of 98.5% (HAC) and 98.8% (HBC) at 800 ppm within the concentration range of our research. The high inhibition efficacy of the HAC and HBC molecules can be explained by the existence of benzene rings, double bonds, and hydroxyl group in addition to the presence of N, O and S atoms^40^. The provided inhibitor's structure and concentration affect the values of the corrosion inhibition efficiency, and surface coverage. Using the following equations, the percentage inhibition efficiency (*η* %) and the degree of surface covering (*θ*) were calculated^41,42^:

1 $$\theta = \frac{{i}_{corr}^{o} - {i}_{corr}}{{i}_{corr}^{o}}$$

2 $$\eta \text{\% }= \frac{{i}_{corr}^{o} - {i}_{corr}}{{i}_{corr}^{o}}\times 100$$

where *i*^o^_corr_ and *i*_corr_ are the corrosion current densities in the absence and presence of the inhibitor, respectively. It can be seen that the values of the inhibition efficiencies calculated from *i*_corr_ rise with rising concentration of HBC and HAC. The *η* % values of HAC and HBC, at each concentration are extremely high, and the maximum values of the efficiencies are 98.5% with HAC and 98.8% with HBC at the 800 ppm within the concentration range of our research.

**Table 1:** Electrochemical parameters for steel dissolution in 0.5 M H_2_SO_4_ solution containing different concentrations of the (***HAC*** and ***HBC***) inhibitors obtained from polarization measurements at 303 K.

Inhibitor name	Conc (ppm)	E_corr_ vs. SCE (mV)	i_corr_ (μA cm^−2^)	β_a_ (mV dec^−1^)	β_c_ (mV dec^−1^)	θ	η_PDP_%
Blank	–	−493.119	2326	231.416	362.743	–	–
***HAC***	100	−563.400	856.868	228.213	290.295	0.6316	63.16
	200	−502.612	199.305	107.946	230.803	0.9143	91.43
	400	−477.552	148.974	106.680	266.700	0.9360	93.60
	600	−535.995	94.995	139.316	226.699	0.9592	95.92
	800	−491.185	34.397	107.630	247.529	0.9852	98.52
***HBC***	100	−545.042	561.494	205.813	292.169	0.7586	75.86
	200	−506.329	166.713	106.240	207.475	0.9283	92.83
	400	−535.041	107.777	165.316	273.690	0.9537	95.37
	600	−526.565	76.317	142.226	271.481	0.9672	96.72
	800	−548.489	28.263	135.770	260.321	0.9878	98.78

E_corr_ is the corrosion potential; i_corr_ is the corrosion current density: β_a_ and β_c_ are Tafel constants for both anode and cathode; k is the corrosion rate; θ is the surface coverage; η_PDP_ is the inhibition efficiency.

Several papers have demonstrated that the ΔE_corr_ value can also be used to determine the corrosion inhibition effect. If the change in E_corr_ is > 85 mV or <  − 85 mV with regard to the *E*_corr_ of the blank solution, the inhibitor compounds can be categorized as anodic or cathodic type inhibitors respectively. When the change in *E*_corr_ is < 85 mV, the chemical inhibitor can be classified as a mixed type^43,44^. The largest displacement displayed by HBC or HAC in the current investigation is ~ 16 mV (see Table 1), leading to conclude that these compounds performed as mixed-type inhibitors. As a result, the anodic dissolution of MS was reduced and the cathodic reaction of H_2_ evolution was delayed when HBC or HAC were added to the 0.5 M H_2_SO_4_ electrolyte. These substances can also serve as a physical barrier to stop the diffusion of corrosive species to the mild steel electrode's surface. Also, the shift of *β*a and *β*_c_ indicate that the metal dissolution processes (Fe → Fe^++^ + 2e^−^) as well as the hydrogen evolution (2H^+^ + 2e^−^ → H_2_) is suppressed by the adsorption of HBC and HAC. Therefore, it confirms that HBC and HAC act as mixed-type corrosion inhibitors which suppress both anodic and cathodic reaction by adsorbing on the MS surface^45^. Tri-thiosemicarbazone derivatives (HBC and HAC) may interact with the d-orbitals of Fe atoms through the interaction of hetero atoms in the HBC and HAC structure that have unshared electron pairs with the steel surface in 0.5M H_2_SO_4_ solution. Since the electron configuration of Fe was [Ar] 4s^2^3d^6^, it is obvious that the electrons in the third orbit were not completely filled. While the filled 4s orbital might interact with the lowest unoccupied molecular orbital of the HBC and HAC, this unfilled iron orbital may bond with the highest occupied molecular orbital of the thiosemicarbazone molecule^46^.

#### Electrochemical impedance measurements, EIS

**Figure 6:**
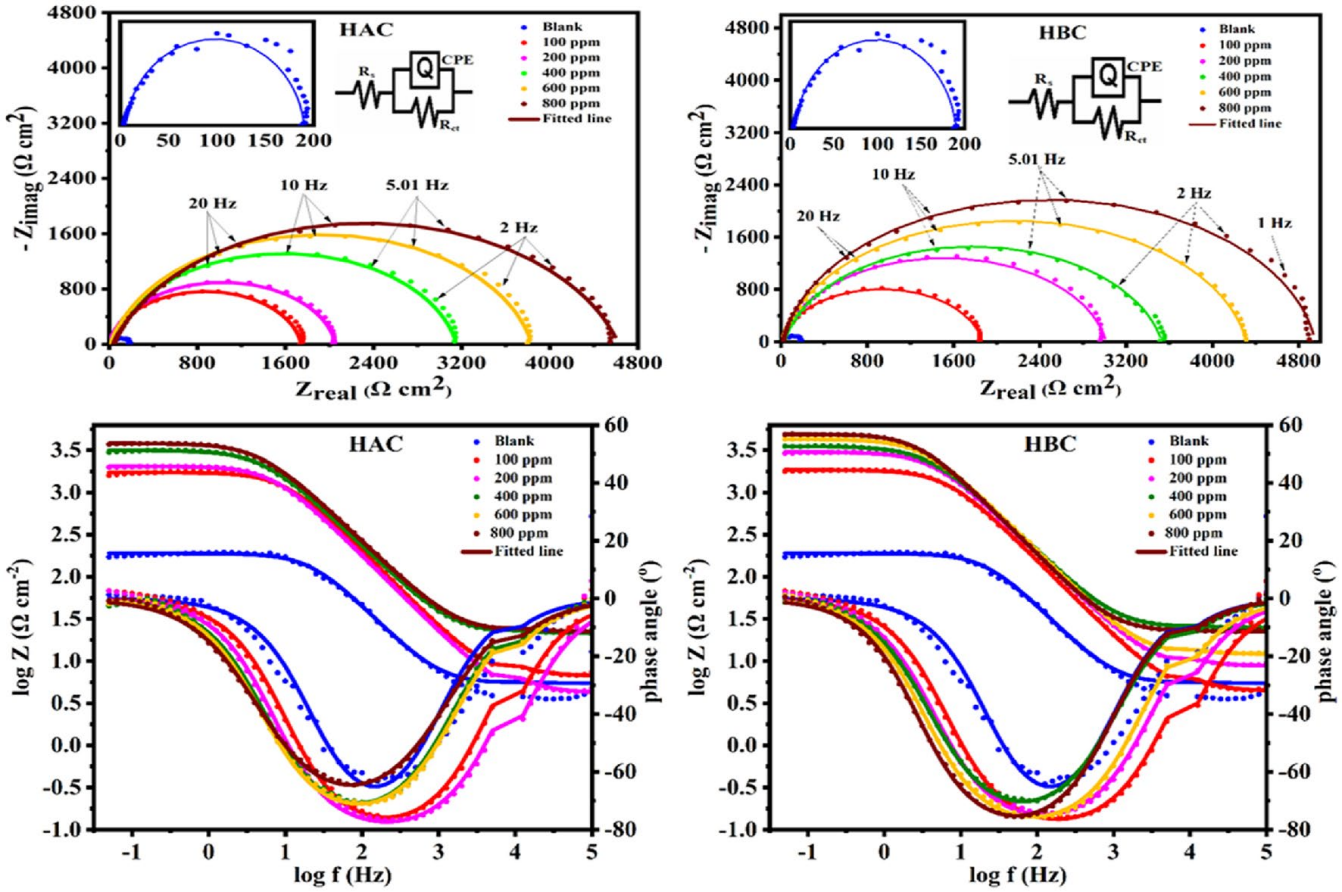
Nyquist, Bode plots and equivalent circuit for steel dissolution in 0.5 M H_2_SO_4_ in the absence and presence of different concentrations of the HAC and HBC inhibitors at 303 K.

EIS is a highly effective method for determining rate of corrosion, due to utilization of a modest ac signal without substantially altering the attributes being recorded or the electrode surface morphology. Additionally, it is feasible to replicate the experimental impedance data using pure electronic models, which can be used to test or validate mechanistic models and to calculate numerical values corresponding to the physical and/or chemical properties of the electrochemical system^47,48^. The results of the EIS measurements obtained with the MS electrode immersed in 0.5 M H_2_SO_4_ in absence and presence of (100–800 ppm) of HAC and HBC inhibitors are presented in Fig. 6 in the form of Bode and Nyquist plots. The presence of a depressed capacitive loop at intermediate frequencies and a single semicircle are the main characteristics of the Nyquist plot. This behavior shows that the corrosion process is under charge transfer control and is related to a single time constant that includes relaxation effects from adsorption events^49,50^. Relaxation processes take place at certain frequencies in the interfacial phenomena governed by diffusion. In an electrochemical system, the characteristic constant of a relaxation process within the time domain is called the relaxation time (τ_dl_), which is defined as the time required for the charge distribution to return to its equilibrium state. This term is frequently used to differentiate between polarization effects that can be attributed to physical processes that underlie the frequency domain overlap^51^. With a rising inhibitor concentrations, the τ_dl_ values increased in the presence of HAC and HBC (Table 2), indicating a slower process of electric charge and discharge at the metal-solution interface because there are more macromolecules adsorbed on the metallic surface^52^. The relaxation of the double layer capacitance is typically connected with the semicircle at high frequencies, and the diameter of this semicircle denotes the charge-transfer resistance.^53,54^. The diameter of the capacitive loop increases after the addition of the investigated inhibitor (HAC and HBC) to the corrosive medium, indicating that the addition of HAC and HBC in the corrosive solution suppresses the corrosion process by blocking the active corrosion centers and flawed or fragile regions by the adsorption of its molecules on the metal surface. The dispersion formula was used to examine the entire set of impedance data using software that came with the electrochemical workstation. On the MS surface, it was discovered that HBC is more efficient than HBC. The data are well-aligned with those from potentiodynamic polarization measurements. Several impedance parameters were calculated using equivalent circuit shown in Fig. 6 and listed in Table 2. The values of *R*_ct_ were given by subtracting the high frequency impedance from the low frequency one as follows^55^: *R*_ct_ = Z_re_(at low frequency) − Z_re_(at high frequency). Increasing the value of *R*_ct_ from (184.6 Ω cm^2^) to (4582 and 4930 Ω cm^2^ for HAC and HBC, respectively) and decreasing the value of double layer capacitance (C_dl_) by increasing the inhibitor concentration indicate that HAC and HBC inhibit corrosion rate of MS in 0.5 M H_2_SO_4_ by adsorption mechanism^56^. At Low-frequency regions (0.01 Hz), as those in the bode plots (Fig. 6), were linked to polarization resistance or charge transfer, and the impedance (|*Z*|_0.01_) might be an indicator of inhibitory strength^57^. With every increase in HAC and HBC concentrations, the corrosion reaction is slowed down by an increase in |*Z*|_0.01_^58^. The insertion of HAC and HBC molecules causes the slopes of bode lines in the middle-frequency band to shift near -1 (pure capacitor), which suggests that the corrosion system's capacitive behavior is becoming more pronounced^59^. Additionally, the height of the peak grew with increasing HAC and HBC concentrations, and all Bode-phase angle curves exhibited a single time constant, indicating a stronger response from HAC and HBC adsorption in the steel/H_2_SO_4_ interface^60^. The data obtained from EIS are in a good agreement with aforementioned results of potentiodynamic polarization measurements.

**Table 2:** Electrochemical parameters calculated from EIS measurements on mild steel electrode in 0.5 H_2_SO_4_ solutions without and with various concentrations of (HAC, HBC) at 303K.

Inhibitor	Conc (ppm)	R_s_ (Ω cm^2^)		R_ct_ (Ω cm^2^)	Y_o_ (μ Ω^−1^ s^n^ cm^−2^)		n	C_dl_ (μF cm^−2^)		Chi squared (χ^2^)	S	α°	τ (ms)	θ	η_z_ (%)
Blank	–		5.469	184.6	28.75	0.9323			19.653	7.79 × 10^–2^	−0.715	−64.18	3.63	–	–
**HAC**	100		6.665	1736	6.89	0.9191			4.669	1.75 × 10^–3^	−0.793	−75.97	8.11	0.894	89.37
	200		4.188	2052	8.12	0.9144			5.536	2.91 × 10^–3^	−0.804	−76.78	11.36	0.910	91.00
	400		21.14	3133	7.59	0.8879			4.736	9.19 × 10^–4^	−0.784	−70.67	14.84	0.941	94.11
	600		22.15	3815	6.97	0.8834			4.321	6.89 × 10^–4^	−0.813	−71.11	16.49	0.952	95.16
	800		42.37	4582	7.95	0.8314			4.063	5.12 × 10^–4^	−0.789	−64.64	18.62	0.960	95.97
**HBC**	100		4.389	1860	9.72	0.9087			6.494	2.61 × 10^–3^	−0.819	−75.73	12.08	0.901	90.08
	200		8.666	3006	8.81	0.8983			5.839	1.21 × 10^–3^	−0.820	−74.11	17.55	0.939	93.86
	400		24.67	3506	9.11	0.8832			5.776	2.26 × 10^–3^	−0.796	−70.05	20.25	0.947	94.73
	600		12.02	4302	7.87	0.9047			5.512	7.34 × 10^–4^	−0.861	−75.61	23.71	0.957	95.71
	800		22.37	4930	7.72	0.92			5.810	5.18 × 10^–4^	−0.864	−75.54	28.64	0.963	96.26

*R*_*s*_ solution resistance, *R*_*ct*_ charge transfer resistant, *Y*_*0*_,* n* constant phase elements, *C*_*dl*_ double layer capacitance, *θ* surface coverage, *η*_*z*_ inhibition efficiency.

### Adsorption isotherm

**Figure 7:**
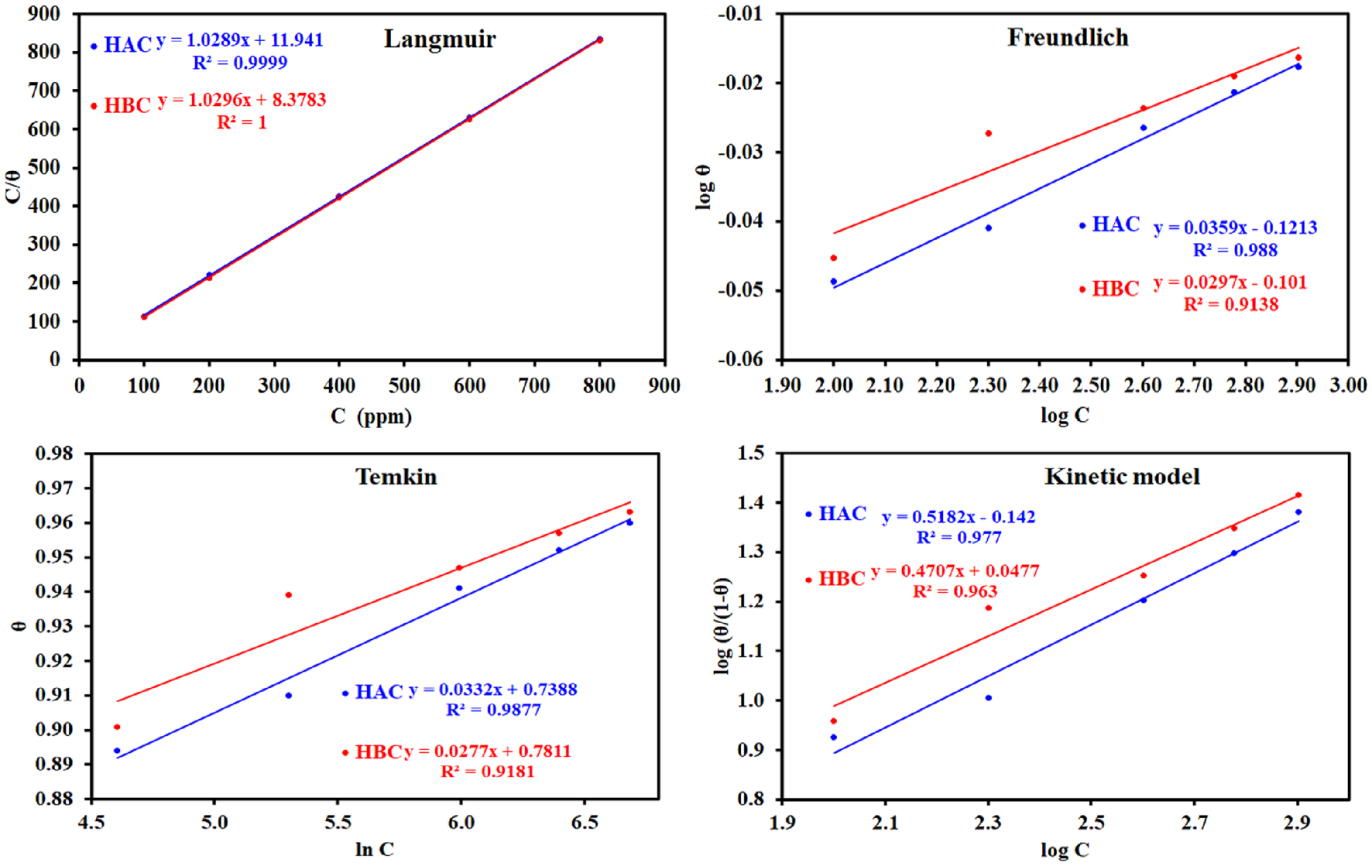
The different adsorption models for ***HAC*** and ***HBC*** compounds on the steel surface in 0.5 M H_2_SO_4_ using electrochemical impedance spectroscopy data at 303 K.

By locating an appropriate isotherm, the adsorption performance of tri-thiosemicarbazone derivatives (HAC and HBC) on the MS surface may be understood. Numerous numerical relations (Freundlich, Langmuir, Temkin, and kinetic-thermodynamic) for the adsorption isotherms were devised and displayed in Fig. 7 to fit the exploratory results of EIS and PDP tests. The data collected in Table 3 show that Langmuir isotherm equation fits our results according to R^2^ values. In Langmuir isotherm equation, the equilibrium constant (K) of the adsorption process and the adsorbate concentration (C) are related as C/θ = 1/K + C^61^. Also, K is linked to the standard free energy of adsorption, ∆G^o^_ads_, by the equation^62^: K = 1/10^6^ exp (− ∆G^o^_ads_/RT), where T stands for the absolute temperature and R for the ideal gas constant while the value 10^6^ is the concentration of water in ppm. Using EIS data, the standard adsorption free energies are equal to −28.56 and −29.45 kJ mol^−1^ for HAC and HBC respectively. The negative sign of ∆G^o^_ads_ indicates the spontaneous nature of the adsorption process of HAC and HBC on MS surface^63^. The obtained value of ∆G^o^_ads_ suggests a physical adsorption process.

**Table 3:** Adsorption isotherms models of the inhibitors with values of R^2^, slopes, intercepts, and thermodynamic parameters (K_ads_ and ΔG^o^_ads_,) by using data obtained from EIS measurements.

Adsorption isotherm model	Linear form equation	Technique	Inhibitor	Slope	Intercept	R^2^	K_ads_ (ppm^−1^)	ΔG^o^_ads_ (kJ/mol)
Freundlich	*log* $\theta$ = $logK$ + *1/n log C*	EIS	HAC	0.03587	−0.12130	0.98796	0.7563	−34.10
			HBC	0.02967	−0.10102	0.91377	0.7925	−34.22
		PDP	HAC	0.18919	−0.53453	0.75559	0.2921	−31.70
			HBC	0.11402	−0.32552	0.80684	0.4726	−32.92
Langmuir	$\frac{c}{\theta }= \frac{1}{\text{K}}+ c$	EIS	HAC	1.02886	11.94111	0.99994	0.0837	−28.56
			HBC	1.02961	8.37830	0.99997	0.1194	−29.45
		PDP	HAC	0.95678	46.54455	0.99783	0.0215	−25.13
			HBC	0.98002	27.77391	0.99959	0.0360	−26.43
Temkin	$\theta$ = $- \frac{1}{2a}\text{ln}C$ $- \frac{1}{2a}\text{ln}K$	EIS	HAC	29.72466	−21.89025	0.98770	0.4788	−32.95
			HBC	33.19060	−25.45033	0.91812	0.4645	−32.87
		PDP	HAC	5.17652	1.21273	0.78113	1.2640	−35.39
			HBC	8.31290	−1.84525	0.82486	0.8009	−34.24
kinetic-thermodynamic	$log\left(\frac{\uptheta }{1-\uptheta }\right)= y \,logK+y \,log \,c$	EIS	HAC	0.51821	−0.14202	0.97695	0.53202749	−33.21
			HBC	0.47065	0.04770	0.96303	1.26285299	−35.39
		PDP	HAC	1.52075	−2.70315	0.92043	0.01669113	−24.49
			HBC	1.36672	−2.17962	0.93930	0.02542296	−25.55

### Reactivity descriptors

**Figure 8:**
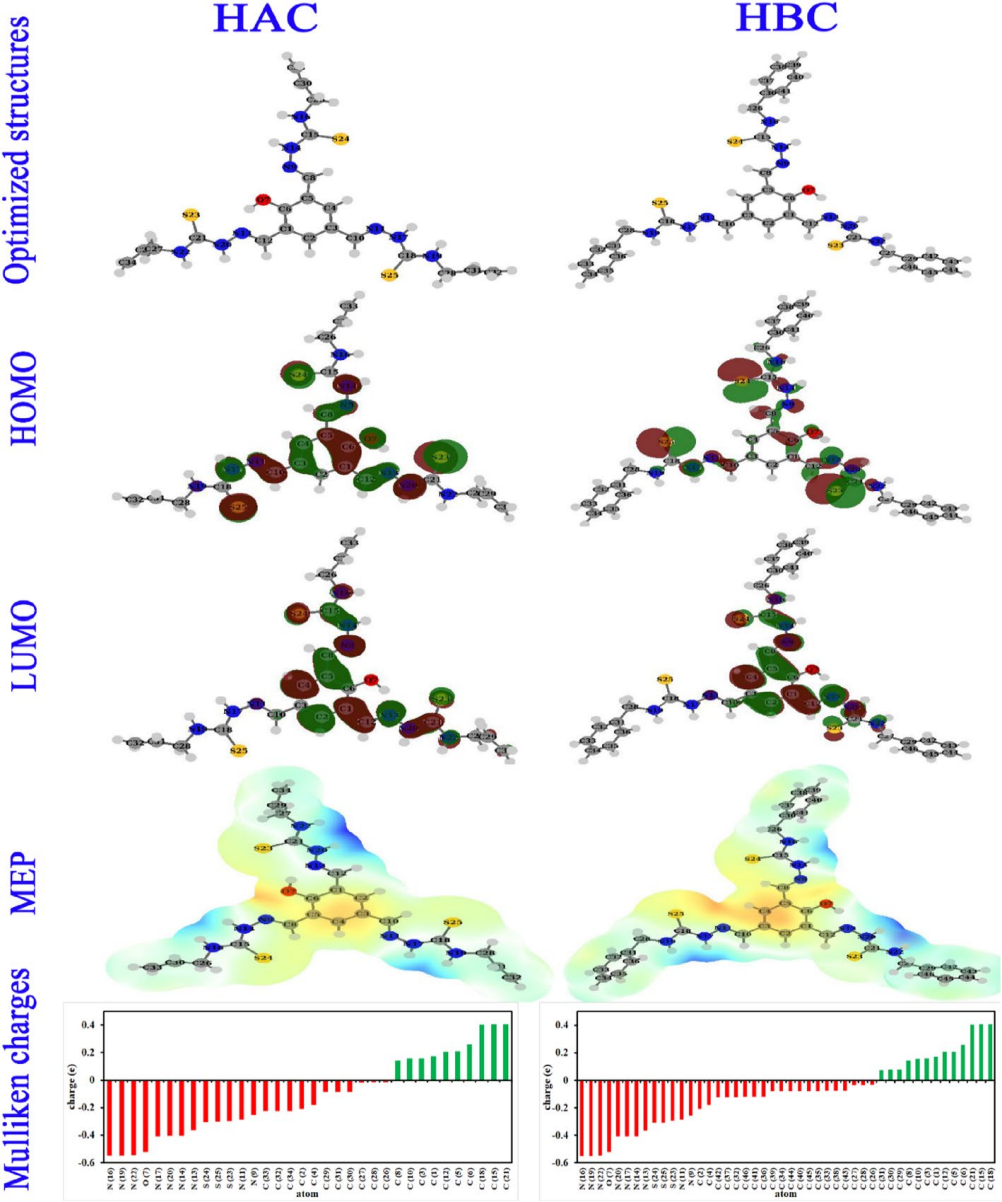
Graphical representation of the optimized molecular structures, highest occupied molecular orbitals (HOMO), lowest unoccupied molecular orbitals (LUMO), molecular electrostatic potential map (MEP), and Mulliken atomic charges (MAC) of HAC and HBC molecules from B3LYP calculations.

After DFT-631G(d,p) and DMol^3^ computations for HAC and HBC molecules, snapshots for the optimized structures, HOMO, LUMO, MEP and Mulliken charges were extracted as shown in Fig. 8 and the extracted quantum parameter are collected in Table 4. The two molecules have a similar skeleton except the terminal groups as –CH=CH_2_ for HAC and benzene moieties for HBC. HAC and HBC structures are roughly planner with a slight difference for the terminal aromatic moieties in case of HBC molecule. This approximate planner geometry with severally distributed active sites may guarantee large surface coverage of steel and enhanced protection. Based on Mulliken atomic charges (MAC), The highly negatively charged atoms are expected to adsorb on steel surface via electron donation and/or electrostatic attraction^64,65^.

**Table 4:** The calculated quantum chemical parameters in eV for the neutral and protonated inhibitors at DFT-631G(d,p) and DMol^3^ in gas phase and in aqueous phase.

Molecule			E_HOMO_ (eV)	E_LUMO_ (eV)	∆E (eV)	∆E _back_ _donation_ (eV)	T.E (eV)	μ (Debye)	M.V (cm^3^/mol)	TNC (e)		σ (eV^−1^)	ε (eV^−1^)	ω (eV)	χ (eV)	η (eV)	∆N (e)	ω + (eV)	ω− (eV)	IE_p_ (%)
		DFT-B3LYP/6-31G(d,p)																		
Gas	**HAC**		−5.3423	−1.5977	3.7446	−0.4681	−2578.9345	2.6848	365.703	−6.56634		0.5341	0.3110	3.2155	3.4700	1.8723	0.3605	1.7146	5.1845	95.97
	**HBC**		−5.1838	−1.5640	3.6198	−0.4525	−3039.9177	9.8065	453.928	−7.13659		0.5525	0.3180	3.1446	3.3739	1.8099	0.3995	1.6839	5.0578	96.26
	**HAC – H**^**+**^		−7.4082	−5.6580	1.7502	−0.2188	−2579.3474	6.0828	368.447	−6.52643		1.1427	0.0410	24.3861	6.5331	0.8751	−0.9788	21.2290	27.7621	95.97
	**HBC – H**^**+**^		−7.3019	−5.5062	1.7956	−0.2245	−3040.3363	7.5510	423.014	−7.00505		1.1138	0.0438	22.8399	6.4041	0.8978	−0.8822	19.7501	26.1542	96.26
Aqueous	**HAC**		−5.7270	−1.8712	3.8558	−0.4820	−70,122.4289	6.6672	406.9090	−6.83204		0.5187	0.2671	3.7433	3.7991	1.9279	0.2648	2.0847	5.8838	95.97
	**HBC**		−5.6846	−1.8756	3.8090	−0.4761	−82,657.2237	19.1639	423.1590	−7.73948		0.5251	0.2666	3.7514	3.7801	1.9045	0.2730	2.0994	5.8795	96.26
	**HAC – H**^**+**^		−5.8698	−2.9322	2.9376	−0.3672	−70,134.9943	11.4344	426.9310	−6.94284		0.6808	0.1517	6.5933	4.4010	1.4688	0.1426	4.5764	8.9774	95.97
	**HBC – H**^**+**^		−5.9802	−2.9346	3.0456	−0.3807	−82,669.3851	16.4024	444.1650	−7.55115		0.6567	0.1533	6.5237	4.4574	1.5228	0.1191	4.4854	8.9428	96.26
DMol^3^																				
Gas	**HAC**		−4.6776	−2.4531	2.2245	−0.2781	−69,699.1095	–	–	−8.455		0.8991	0.1750	5.7144	3.5653	1.1122	0.5640	4.0708	7.6361	95.97
	**HBC**		−4.5736	−2.3461	2.2275	−0.2784	−82,123.5153	–	–	−10.369		0.8979	0.1861	5.3741	3.4599	1.1137	0.6106	3.7833	7.2432	96.26
	**HAC – H**^**+**^		−6.8073	−5.7377	1.0696	−0.1337	−69,710.7588	–	–	−8.392		1.8699	0.0272	36.7836	6.2725	0.5348	−1.3579	33.7142	39.9867	95.97
	**HBC – H**^**+**^		−6.7374	−5.7147	1.0227	−0.1278	−82,134.8161	–	–	−10.156		1.9557	0.0264	37.9040	6.2260	0.5113	−1.3749	34.8549	41.0810	96.26
Aqueous	**HAC**		−5.0831	−2.7723	2.3107	−0.2888	−69,700.6955	–	–	−9.082		0.8655	0.1498	6.6762	3.9277	1.1554	0.3861	4.8568	8.7845	95.97
	**HBC**		−5.0400	−2.7887	2.2513	−0.2814	−82,125.1389	–	–	−11.068		0.8884	0.1469	6.8058	3.9144	1.1257	0.4023	4.9894	8.9037	96.26
	**HAC – H**^**+**^		−5.2349	−3.5632	1.6716	−0.2090	−69,713.0441	–	–	−8.797		1.1964	0.0864	11.5767	4.3991	0.8358	0.2518	9.4816	13.8807	95.97
	**HBC – H**^**+**^		−5.2219	−3.6040	1.6179	−0.2022	−82,137.0643	–	–	−10.853		1.2361	0.0831	12.0362	4.4129	0.8090	0.2516	9.9309	14.3438	96.26

MAC diagrams in Fig. 8 show several negatively charged atoms like N, O, S and C atoms. The three azomethane nitrogen atoms (C=**N**) C_16_, C_19_ and C_22_ showed the highest negative charges with values ranged from −0.5468 to −0.5505 e followed by the phenolic oxygens of the central aromatic moieties by the value of −0.5214 e followed by the diazines nitrogen’s (N–N) with the values ranged from −0.3627 e to −0.4087 e and the sulfur atoms of C=**S** groups (S_23_, S_24_ and S_25_) by values ranged from −0.2972 e to −0.3089 e at the 631-G(d,p) basis set. The summation of MAC (TNC) is higher for MBC molecule than HAC molecule to confirm the priority of HBC molecule in electron donation and/or electrostatic attraction and so steel surface protection. Figure 8 also, showed the molecular electrostatic potential (MEP) map which divide the HAC and HBC molecules into through main regions; low potential (red spots), moderate potential (yellow or green spots) and high potential sections (blue spots), which correspond to electron-rich (where the steel prefers to bind), electron-moderate and electron-poor regions, respectively^66^. It is important to keep in mind that the quantity of electrophile and nucleophile sites is not always a good indicator of the molecule's overall reactivity, but it does point to potential places of enhanced steel interactions^67^. MEP of HAC and HAB molecules showed extremely electron-rich region around the central aromatic moiety and phenolic oxygen then the sulfur atoms and the nitrogen atoms while the blue region are around the hydrogens attached to the nitrogen atoms of the diazene’s and the moderate-electrons regions (green) are around the terminal groups either benzene moieties or –CH=CH_2_ groups. The graphical representations of the frontiers molecular orbitals (FMO) either the electron donating orbitals (HOMO) or the electron accepting orbitals (LUMO) for HAC and HBC molecules are shown in Fig. 8. Chemical reactivity and so the adsorption ability between the HAC or HBC molecules and steel is a function of the donating power of HOMO orbitals to the empty d-orbitals in steel^68^. HOMO orbitals are mostly distributed on large areas of HAC and HBC molecules including the central aromatic moieties, phenolic oxygen, sulfur atoms and the nitrogen atoms. These active sites can share their electrons with steel forming a stable and coherent protective layer from corrosion^69^. A quantitative analysis of E_HOMO_ values in Table 4, revealed that HBC possesses higher E_HOMO_ values than HAC molecule either in DFT-631-G data or DMol^3^ data. This suggests that the electron donating power of HBC molecule is higher than HAC molecule^70^. Also, the calculated molecular volume of HBC molecule is higher than HAC molecule which guarantee that a single HBC molecule will occupy more surface area on steel surface and higher corrosion protection^71^.

The reactivity of the organic molecules (HAC and HBC) towards the steel metallic surface enhanced by the reduction of the energy gap between HOMO and LUMO (ΔE = E_LUMO_ − E_HOMO_)^72^. Low ΔE values of HAC and HBC molecules (Table 4) suggest high reactivity and enhance protection. The binding of HAC and HBC molecule to steel surface can be enhanced by the electron retro donation as indicated from positive values of hardness (η) and negative values of ΔE_back-donation_^73^. The relationship between dipole moment (**μ**) and the efficiency is very favorable since the buildup and accumulation of HAC and HBC on the metallic surface is more advantageous by large **μ** values^74^. The μ values of HBC are higher than that of HAC, which confirms the predominance of HBC in protection efficiency. Also, electronegativity (**χ**) of HBC molecules is lower than HAC molecule which indicate lower electron attracting ability and higher protection for HBC molecule^75^. The tendency of electrons for following from HAC and HBC molecules to steel surface (**∆N)** was calculated to confirm the ability of the studied compounds to donate electrons to the steel empty orbitals^76^. Also, the electron donating power (ω −) values are higher than electron receiving power (ω +) for both HAC and HBC molecules as indication of the electron follow from the organic molecules to the steel surface is the favorable mode. The Natural bond orbitals (NBOs) were done to determine which atoms or groups in HAC or HBC HOMO possess the priority in sharing electrons to the vacant d-orbitals of steel^77^. The orbital hybridization pattern for the proposed locations of interactions of HAC and HBC molecules are listed in Table 5, accompanied by the appropriate eigenvalues in eV (ordered by the priority of electron donating capacity). The supplementary Tables S1and S2 include the matching surface densities. The hybridizations of HAC in order of donating ability were found as LP (1) C1 > (LP (2) S24 > LP (2) S23 > LP (2) S25 > BD (2) C4-C5 > BD (2) C2-C3 > LP (1) N16 > LP (1) N14 > LP (1) N19 > LP (1) N17 > LP (1) N22 > BD (2) C30-C33 > BD (1) C21-S23 > BD (2) C31-C32 > BD (2) C29-C34 > LP (1) N20 > LP (2) O7 > BD (2) C8-N9 > BD (2) C10-N11 > BD (2) C12-N13 > LP (1) N9 > LP (1) N11 > BD (1) C15-S24 > BD (1) C18-S25. This ranking shows the priority of the central aromatic moiety then the lone pairs of the sulfur hetero atoms followed by the lone pairs of nitrogen atoms (C-**N**H) then the π bonds of the terminal -CH = CH_2_ groups in donating ability. Similar trend obtained for HBC molecule as LP (1) C1 > LP (2) S25 > LP (2) S24 > LP (2) S23 > BD (2) C4-C5 > BD (2) C33-C34 > BD (2) C38-C39 > BD (2) C31-C32 > BD (2) C35-C36 > BD (2) C30-C37 > BD (2) C40-C41 > BD (2) C2-C3 > BD (2) C43-C44 > BD (2) C45-C46 > BD (2) C29-C42 > LP (1) N14 > LP (1) N16 > LP (1) N19 > LP (1) N22 > LP (1) N17 > BD (1) C18-S25 > LP (2) O7 > BD (1) C21-S23 > LP (1) N20. It is noted that the donating ability of the terminal aromatic moieties overcomes the donating ability of lone pairs of nitrogen atoms which clarify their valuable role in adsorption and steel protection.

**Table 5:** NBOs at expected inhibitor-metal interactions ordered according to their energies (highest to lowest).

Bond	Occupancy	Energy (eV)	NBO	s % (atom 1)	p % (atom 1)	s % (atom 2)	p % (atom 2)
HAC							
LP (1) C_1_	1.10704	−3.1296	P	0	100	–	–
LP (2) S_24_	1.86720	−5.0032	sp^99.99^	0.02	99.94	–	–
LP (2) S_23_	1.86607	−5.1267	sp^99.99^	0.06	99.90	–	–
LP (2) S_25_	1.86818	−5.1560	sp^99.99^	0.02	99.94	–	–
BD (2) C_4_-C_5_	1.66305	−6.5857	0.6732 p^1.00^ + 0.7394 p^1.00^	0	99.95	0	99.97
BD (2) C_2_-C_3_	1.65440	−6.6722	0.6825 p^1.00^ + 0.7309 p^1.00^	0	99.96	0	99.98
LP (1) N_16_	1.71265	−7.1667	sp^65.77^	1.50	98.49	–	–
LP (1) N_14_	1.64935	−7.2121	p^1.00^	0	99.99	–	–
LP (1) N_19_	1.71363	−7.3193	sp^58.14^	1.69	98.29	–	–
LP (1) N_17_	1.65358	−7.4079	p^100^	0	100	–	–
LP (1) N_22_	1.70791	−7.5403	sp^42.95^	2.27	97.70	–	–
BD (2) C_30_-C_33_	1.97388	−7.5814	0.7073 sp^99.99^ + 0.7069 sp^99.99^	0.29	99.64	0.26	99.67
BD (1) C_21_-S_23_	1.98728	−7.6273	0.5437 sp^99.99^ + 0.8393 sp^99.99^	0.41	99.44	0.16	99.60
BD (2) C_31_-C_32_	1.97368	−7.6768	0.7080 sp^99.99^ + 0.7062 sp^99.99^	0.29	99.65	0.26	99.67
BD (2) C_29_-C_34_	1.97226	−7.6814	0.7043 sp^99.99^ + 0.7099 sp^99.99^	0.50	99.43	0.46	99.47
LP (1) N_20_	1.67993	−7.9359	sp^99.99^	0.69	99.30	–	–
LP (2) O_7_	1.77582	−8.0064	P^1.00^	0	99.87		
BD (2) C_8_-N_9_	1.92485	−8.4702	0.6500 P^1.00^ + 0.7599 P^1.00^	0	99.84	0	99.82
BD (2) C_10_-N_11_	1.93178	−8.7068	0.6405 P^1.00^ + 0.7680 P^1.00^	0	99.83	0	99.83
BD (2) C_12_-N_13_	1.93877	−9.5138	0.6406 P^1.00^ + 0.7679 P^1.00^	0	99.83	0	99.83
LP (1) N_9_	1.92676	−9.6930	sp^1.97^	33.63	66.29	–	–
LP (1) N_11_	1.92702	−10.0168	sp^2.03^	32.99	66.94	–	–
BD (1) C_15_-S_24_	1.94548	−10.4323	0.6190 sp^7.86^ + 0.7854 sp^17.96^	11.27	88.59	5.26	94.38
BD (1) C_18_-S_25_	1.94439	−10.7536	0.6236 sp^7.43^ + 0.7817 sp^17.05^	11.85	88.02	5.52	94.11
HBC							
LP (1) C_1_	1.10685	−3.0959	p	0	100	–	–
LP (2) S_25_	1.87111	−4.6427	sp^99.99^	0.09	99.86	–	–
LP (2) S_24_	1.86980	−4.9108	sp^99.99^	0.02	99.94	–	–
LP (2) S_23_	1.85855	−5.3330	sp^99.99^	0.03	99.93	–	–
BD (2) C_4_-C_5_	1.65956	−6.5786	0.6715 p^1.00^ + 0.7410 p^1.00^	0	99.95	0	99.97
BD (2) C_33_-C_34_	1.66686	−6.6716	0.7063 p^1.00^ + 0.7079 p^1.00^	0	99.96	0	99.96
BD (2) C_38_-C_39_	1.66613	−6.6868	0.7063 p^1.00^ + 0.7079 p^1.00^	0	99.96	0	99.96
BD (2) C_31_-C_32_	1.66480	−6.6893	0.7101 p^1.00^ + 0.7041 p^1.00^	0.01	99.96	0	99.96
BD (2) C_35_-C_36_	1.67192	−6.6977	0.7067 p^1.00^ + 0.7075 p^1.00^	0	99.96	0	99.96
BD (2) C_30_-C_37_	1.66490	−6.7127	0.7107 p^1.00^ + 0.7035 p^1.00^	0.01	99.96	0	99.96
BD (2) C_40_-C_41_	1.67151	−6.7170	0.7065 p^1.00^ + 0.7077 p^1.00^	0	99.96	0	99.96
BD (2) C_2_-C_3_	1.65690	−6.7336	0.6805 p^1.00^ + 0.7327 p^1.00^	0	99.96	0	99.98
BD (2) C_43_-C_44_	1.66204	−6.9481	0.7069 p^1.00^ + 0.7074 p^1.00^	0	99.96	0	99.96
BD (2) C_45_-C_46_	1.67015	−7.0039	0.7054 p^1.00^ + 0.7088 p^1.00^	0	99.96	0	99.96
BD (2) C_29_-C_42_	1.66624	−7.0177	0.7129 p^1.00^ + 0.7013 p^1.00^	0.01	99.96	0	99.96
LP (1) N_14_	1.65421	−7.1822	sp^99.99^	0.05	99.95	–	–
LP (1) N_16_	1.71863	−7.2198	sp^39.20^	2.49	97.49	–	–
LP (1) N_19_	1.72103	−7.2557	sp^31.57^	3.07	96.91	–	–
LP (1) N_22_	1.69087	−7.3712	sp^1.00^	0	99.99	–	–
LP (1) N_17_	1.67365	−7.4131	sp^99.99^	0.37	99.63	–	–
BD (1) C_18_-S_25_	1.97917	−7.5953	0.5660 sp^49.65^ + 0.8244 sp^99.99^	1.97	97.88	0.86	98.87
LP (2) O_7_	1.78205	−8.1075	sp^99.99^	0.04	99.84	–	–
BD (1) C_21_-S_23_	1.97939	−8.1956	0.5448 sp^46.54^ + 0.8386 sp^86.22^	2.10	97.74	1.14	98.61
LP (1) N_20_	1.72298	−8.3974	Sp^13.69^	6.81	93.16	–	–

The reactivity of individual atoms for donating or accepting electrons were estimated from Fukui functions (${f}^{+}$ and ${f}^{-}$). It is calculated from finite difference between neutral, cationic and anionic forms^78^. In case of HAC, the highest values of ${f}^{-}$ (Table 6) were attributed to the hetero atoms firstly sulfur atoms S_23_, S_24_ and S_25_ then the nitrogen atoms and phenolic oxygen atom as the most active sites for electron sharing with the metallic surface. A close situation is obtained for HBC molecule. So, the most effective active sites for HAC and HBC molecules to engage with steel surfaces and create bonds are the lone electron pairs found on most heteroatoms^79^.

**Table 6:** condensed Fukui functions for local reactivities in *HAC* and *HBC* molecules calculated by DMol3 method.

Atom	HAC						Atom	HBC					
	Gas phase			Aqueous phase				Gas phase			Aqueous phase		
	$f^{ + }$	$f^{ - }$	$\Delta f$	$f^{ + }$	$f^{ - }$	$\Delta f$		$f^{ + }$	$f^{ - }$	$\Delta f$	$f^{ + }$	$f^{ - }$	$\Delta f$
C (1)	−0.002	0.01	−0.012	0.014	0.008	0.006	C (1)	0.001	0.008	−0.007	0.016	0.007	0.009
C (2)	0.027	0.012	0.015	0.053	0.011	0.042	C (2)	0.023	0.014	0.009	0.046	0.017	0.029
C (3)	0.003	0.008	−0.005	0.012	0.002	0.01	C (3)	0.003	0.006	−0.003	0.006	0.006	0
C (4)	0.054	0.004	0.05	0.077	0.014	0.063	C (4)	0.053	0.005	0.048	0.08	0.013	0.067
C (5)	0.012	0.008	0.004	0.023	0.003	0.02	C (5)	0.013	0.007	0.006	0.02	0.007	0.013
C (6)	0.023	0.021	0.002	0.03	0.036	−0.006	C (6)	0.025	0.019	0.006	0.044	0.023	0.021
O (7)	0.024	0.021	0.003	0.019	0.028	−0.009	O (7)	0.024	0.02	0.004	0.025	0.02	0.005
C (8)	0.007	0.015	−0.008	0.025	0.019	0.006	C (8)	0.006	0.015	−0.009	0.023	0.016	0.007
N (9)	0.018	0.034	−0.016	0.038	0.024	0.014	N (9)	0.021	0.034	−0.013	0.033	0.033	0
C (10)	0.007	0.012	−0.005	0.039	0	0.039	C (10)	0.006	0.011	−0.005	0.02	0.015	0.005
N (11)	0.017	0.029	−0.012	0.021	0.035	−0.014	N (11)	0.02	0.027	−0.007	0.027	0.036	−0.009
C (12)	0.079	−0.005	0.084	0.078	0.003	0.075	C (12)	0.06	0.002	0.058	0.068	0.007	0.061
N (13)	0.052	0.005	0.047	0.072	−0.01	0.082	N (13)	0.047	0.013	0.034	0.053	0.011	0.042
N (14)	0.006	0.003	0.003	−0.003	0.017	−0.02	N (14)	0.003	0.007	−0.004	0.006	0.012	−0.006
C (15)	0	0.017	−0.017	0.008	0.014	−0.006	C (15)	0.003	0.013	−0.01	0.009	0.011	−0.002
N (16)	0.009	0.013	−0.004	0.008	0.013	−0.005	N (16)	0.011	0.013	−0.002	0.009	0.01	−0.001
N (17)	0.001	0.013	−0.012	−0.029	0.049	−0.078	N (17)	0.003	0.012	−0.009	0.004	0.02	−0.016
C (18)	0.003	0.009	−0.006	0	0.016	−0.016	C (18)	0.003	0.007	−0.004	0.005	0.009	−0.004
N (19)	0.01	0.012	−0.002	0.019	0	0.019	N (19)	0.01	0.013	−0.003	0.007	0.011	−0.004
N (20)	−0.004	0.012	−0.016	−0.008	0.026	−0.034	N (20)	0.002	0.005	−0.003	0.011	0.007	0.004
C (21)	0.013	0.007	0.006	0.024	0.002	0.022	C (21)	0.014	0.001	0.013	0.018	0.005	0.013
N (22)	0.021	0.008	0.013	0.02	0.006	0.014	N (22)	0.02	0.008	0.012	0.018	0.005	0.013
S (23)	0.096	0.042	0.054	0.066	0.086	−0.02	S (23)	0.085	0.042	0.043	0.075	0.073	0.002
S (24)	0.043	0.16	−0.117	0.03	0.181	−0.151	S (24)	0.044	0.166	−0.122	0.04	0.175	−0.135
S (25)	0.032	0.152	−0.12	0.01	0.177	−0.167	S (25)	0.037	0.142	−0.105	0.03	0.175	−0.145
C (26)	0.001	−0.029	0.03	0.024	−0.039	0.063	C (26)	−0.009	−0.02	0.011	−0.004	−0.013	0.009
C (27)	−0.008	−0.016	0.008	0.025	−0.038	0.063	C (27)	−0.014	−0.008	−0.006	−0.007	−0.006	−0.001
C (28)	0.004	−0.03	0.034	0.035	−0.048	0.083	C (28)	−0.008	−0.016	0.008	−0.003	−0.013	0.01
C (29)	−0.015	0	−0.015	−0.019	0.017	−0.036	C (29)	−0.005	−0.003	−0.002	−0.003	−0.001	−0.002
C (30)	−0.016	−0.001	−0.015	−0.016	0.014	−0.03	C (30)	−0.003	−0.006	0.003	−0.002	−0.003	0.001
C (31)	−0.018	0.002	−0.02	−0.03	0.029	−0.059	C (31)	−0.003	−0.005	0.002	−0.001	−0.003	0.002
C (32)	0.012	0.011	0.001	0.034	−0.023	0.057	C (32)	0.001	0.001	0	0.001	0.003	−0.002
C (33)	0.013	0.012	0.001	0.009	0.004	0.005	C (33)	0.002	0.002	0	0.001	0.001	0
C (34)	0.02	0.006	0.014	0.014	−0.003	0.017	C (34)	0.004	0.005	−0.001	0.001	0.003	−0.002
							C (35)	0.002	0.002	0	0.001	0.001	0
							C (36)	−0.001	−0.001	0	0.001	0.002	−0.001
							C (37)	0.001	0.001	0	0.001	0.003	−0.002
							C (38)	0.002	0.003	−0.001	0.001	0.001	0
							C (39)	0.004	0.006	−0.002	0.001	0.003	−0.002
							C (40)	0.002	0.003	−0.001	0.001	0.001	0
							C (41)	−0.001	−0.001	0	0.001	0.003	−0.002
							C (42)	0.003	0	0.003	0.003	0.001	0.002
							C (43)	0.004	0.001	0.003	0.001	0.001	0
							C (44)	0.007	0.003	0.004	0.002	0.001	0.001
							C (45)	0.004	0.001	0.003	0.002	0.001	0.001
							C (46)	−0.001	−0.001	0	0.001	0.001	0

### Molecular dynamics and Monte Carlo simulations

**Figure 9:**
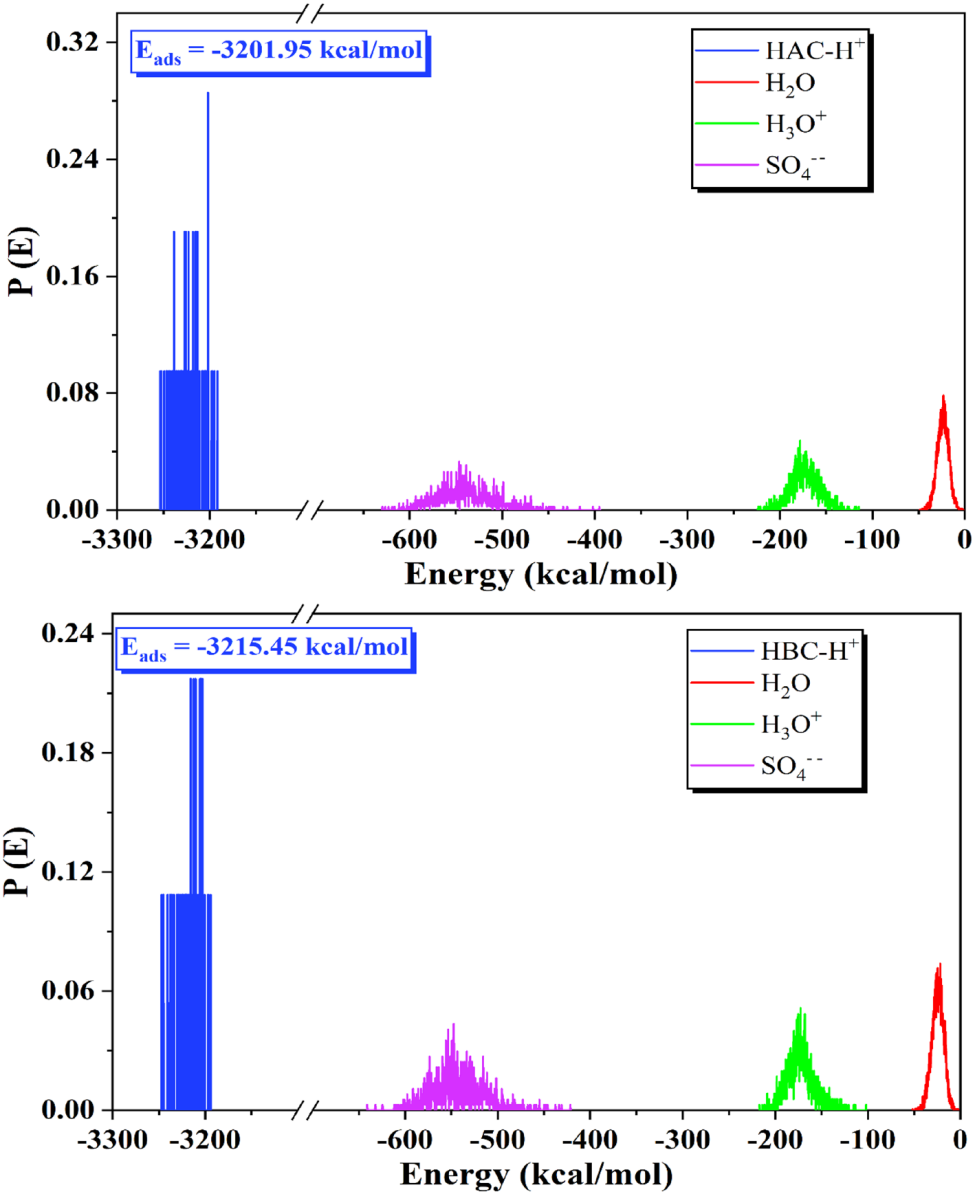
Distribution of the *E*_ads_ of the (HAC-H^+^ and HBC-H^+^/200 H_2_O/19 H_3_O^+^/10 SO_4_^--^) system via MC simulation.

The previously discussed data focused on the HAC and HBC molecules themselves but their adsorption on steel surface was investigated via MC and MD simulations. Details about how HAC and HBC molecules interact with the surface of steel in the aggressive solution can be provided via MC and MD modeling^80,81^. For MC calculations, the different forms of energies like electrostatic, Van der Waals, intramolecular, average and total energies during the optimization process of (HAC-H^+^ and HBC-H^+^/200H_2_O/19 H3O^+^/10 SO_4_^--^) systems onto the Fe (110) surface were calculated as shown in Figure S1. For MD calculations, the most stable adsorption sites of (HAC-H^+^ and HBC-H^+^/Fe (110) were discovered via the examination of T (K) fluctuations Figure S2 shows how little T(K) variation there is, indicating that the MD calculations in our system were done effectively^82^. Figure 9 displays, by MC modeling, the distribution of the adsorption of the (HAC-H^+^ and HBC-H^+^/200 H_2_O/19 H_3_O^+^/10 SO_4_^--^)/Fe (110). E_ads_ are distributed differently in the HAC-H^+^ or HBC-H^+^ molecules, H_3_O^+^ ions, SO_4_^--^ ions, and H_2_O molecules. Compared to the water molecules and dangerous corrosive ions (H_3_O^+^ ions, SO_4_^--^ions), the HAC-H^+^ and HBC-H^+^ molecule have a substantially larger E_ads_ distribution. The E_ads_ distribution in Fig. 9 shows that the HAC-H^+^ and HBC-H^+^ molecules may progressively replace the adsorbed H_3_O^+^ ions, SO_4_^--^ ions, and H_2_O molecules from the steel surface^83^.

**Figure 10:**
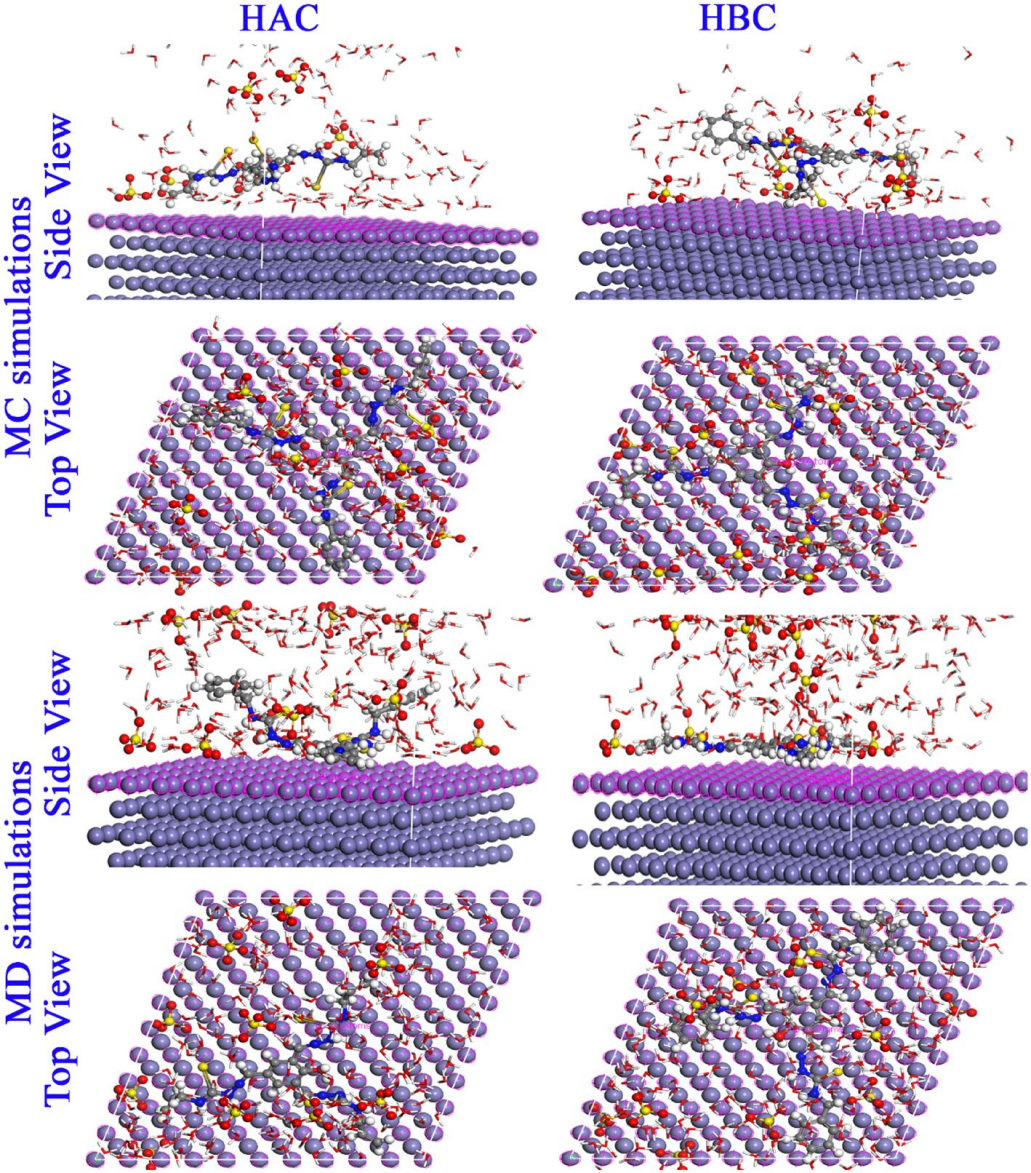
MC and MD simulations results for the most favorable modes of adsorption configurations and positions obtained for (HAC-H^+^ and HBC-H^+^/200 H_2_O/19 H_3_O^+^/10 SO_4_^--^) on Fe (1 1 0) surface, side, and top view.

**Figure 11:**
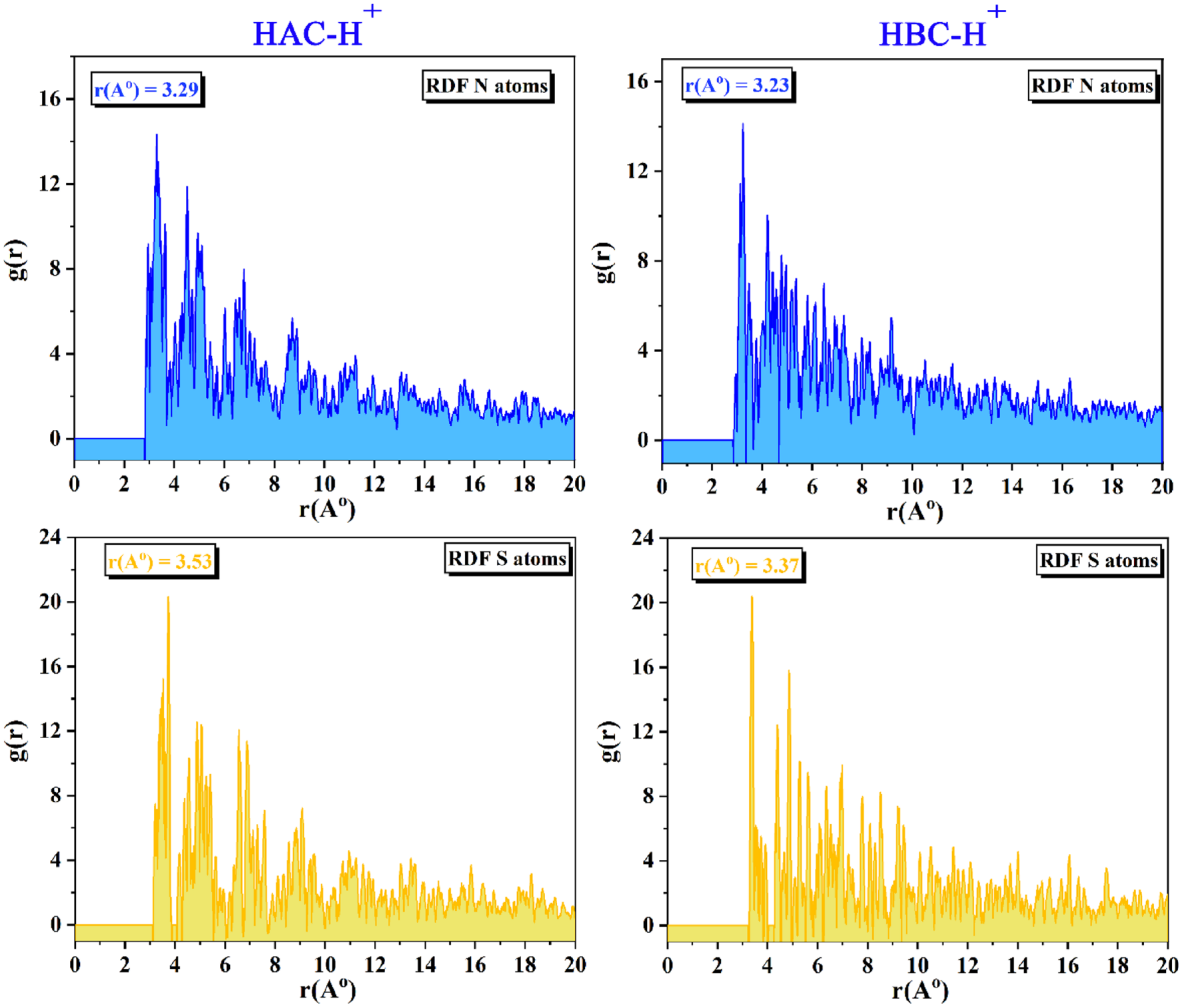
RDF of the O, N and S heteroatoms for HAC and HBC inhibitors/Fe (110), obtained via MD.

The most stable equilibrium configuration for (HAC-H^+^ and HBC-H^+^/200 H_2_O/19 H_3_O^+^/10 SO_4_^--^) on Fe (1 1 0) surface, side, and top view as obtained from MC and MD simulations are collected in Fig. 10. The HAC-H^+^ and HBC-H^+^ orientation during adsorption on the steel surface is obviously flat, which suggests that the steel surface and the active sites in the HAC-H^+^ and HBC-H^+^ molecules interact strongly with maximum surface coverage^84^. The hetero atoms (S, N, O) lone pairs, double bonds (C=S, C=N, C=C) and the aromatic moieties tightly held the HAC-H^+^ and HBC-H^+^ molecules to the surface and shield the other corrosive ions from interaction with steel^85^. The MC adsorption and binding energies are shown in Table 7. For the gas or aqueous phase adsorption, HAC and HBC molecules are listed in order of their effectiveness as inhibitors: HBC (neutral or protonated) > HAC (neutral or protonated). The extra high values of binding energies demonstrates the excellent effectiveness of HAC and HBC molecules in preventing corrosion in steel^86^. The bond length between the hetero atoms (N, S) of HAC and HBC molecules and steel surface were examined using radial distribution functions (RDF)^87,88^ as shown in Fig. 11. The type of adsorption mode can be indicated by peaks in the RDF graph that appear at definite distances from steel surface. For physisorption, the RDF peaks are expected to be present at distances higher than 3.5 Å, but the chemisorption process is implicated when the peak is present between 1 and 3.5 Å^59,89^. RDF peak positions for the S and N atoms are found at values less than 3.5 Å as indication of chemisorption and HBC molecule is slightly close to surface than HAC molecule.

**Table 7:** the outputs and descriptors calculated by the Monte Carlo simulations for adsorption of ***HAC*** and ***HBC*** on Fe (110) (in kcal/ mol).

Phase	Inhibitor	Total energy (kcal mol^−1^)	Adsorption energy (kcal mol^−1^)	Rigid adsorption energy (kcal mol^−1^)	Deformation energy (kcal mol^−1^)	(dE_ads/dNi_) (kcal mol^−1^)	Binding energy (kcal mol^−1^)	IE* (%)
**Gas phase**	***HAC***	−575.985	−2703.807	−275.561	−2428.245	−2703.807	2703.807	95.97
	***HBC***	−591.033	−2792.673	−364.235	−2428.438	−2792.673	2792.673	96.26
	***HAC*** − **H**^**+**^	−571.761	−3390.063	−270.838	−3119.225	−3390.063	3390.063	95.97
	***HBC ***− **H**^**+**^	−588.264	−3481.631	−360.374	−3121.257	−3481.631	3481.631	96.26
**Aqueous phase**	***HAC***	−7943.246	−1023.703	−7845.544	−2391.486	−2557.094	2557.094	95.97
	***HBC***	−7759.114	−1.12.672	−7736.428	−2390.228	−2585.535	2585.535	96.26
	***HAC ***− **H**^**+**^	−7611.257	−1060.154	−7516.947	−3084.595	−3201.951	3201.951	95.97
	***HBC ***− **H**^**+**^	−7690.809	−1075.616	−7670.715	−3085.444	−3215.453	3215.453	96.26

### Inhibition mechanism

**Figure 12:**
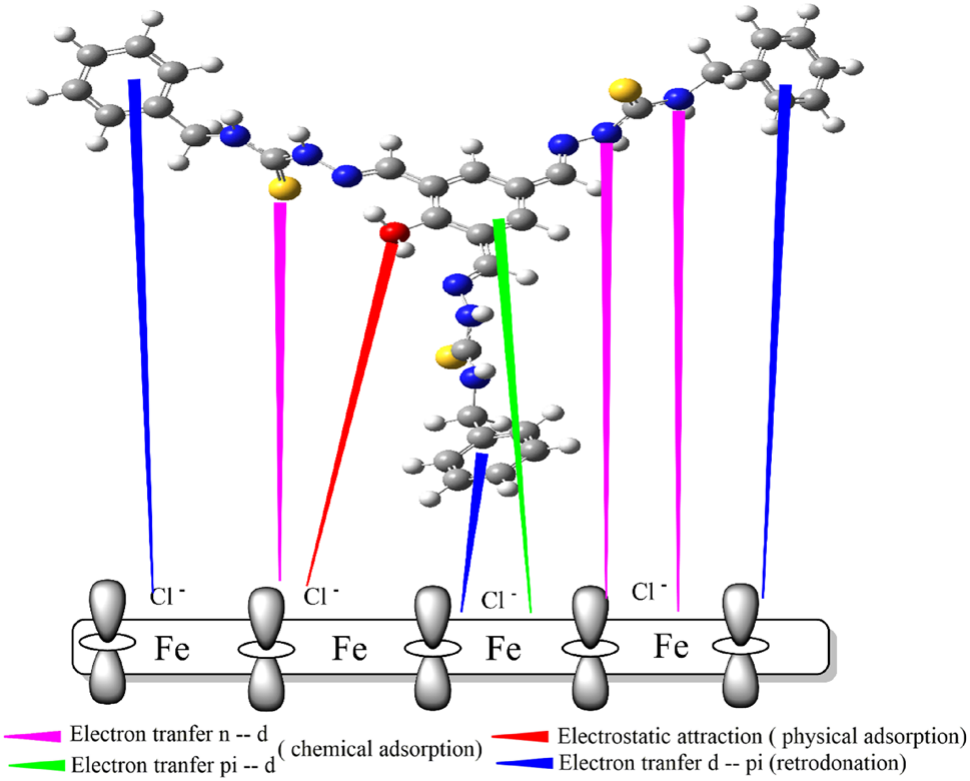
Possible adsorption mechanism of inhibitor (HBC) on mild steel alloy surface in 0.5 M H_2_SO_4_.

The synthesized organic inhibitors HAC and HBC exhibited promising corrosion inhibition properties. The adsorption process, influenced by various factors such as the chemical structure of inhibitors, plays a crucial role in the inhibitory mechanism of corrosion inhibitors. As illustrated in Fig. 12, the proposed adsorption mechanism of HBC on the mild steel surface involves both donation and back donation. The protonated sites of the organic inhibitors are thought to physically adsorb at negatively charged sites on Cl^-^ ions, preventing corrosive ions from attacking the steel alloy surface and causing dissolution. Key components of Tri-thiosemicarbazone derivatives (HAC and HBC) as corrosion inhibitors include the presence of pi electrons in aromatic rings and other functional groups that interact with the metal surface by donating electrons. These substances can also act as a physical barrier, halting the diffusion of corrosive species to the surface of the mild steel electrode. As a result, back donation from the metal surface to the acceptor sites reduces electron repulsion on the CS surface. Consequently, the study anticipates the occurrence of three different types of adsorptions—physisorption, chemisorption, and back donation—which collectively contribute to enhanced protection. The adsorption of HBC and HAC molecules on active sites and/or the deposition of corrosion products on the alloy surface are the two primary mechanisms for corrosion suppression^90^. The orientation of HAC-H^+^ and HBC-H^+^ during adsorption on the steel surface (as indicated from MD/MC simulations) appears to be flat, indicating strong interaction between the steel surface and the active sites in the HAC-H^+^ and HBC-H^+^ molecules, resulting in maximum surface coverage.

The HBC and HAC inhibitors assessed in comparison to other thiosemicarbazones inhibitors in literature, considering their structural features, concentration, and effectiveness in inhibiting a specific process. Furthermore, Table 8 provides data regarding thiosemicarbazones inhibitors reported in literature with structural similarities to HBC and HAC, along with their respective inhibition efficiencies. The inhibition efficiency of HBC and HAC against mild steel alloy corrosion was found to be comparable to, and in some instances even superior to, several other inhibitors^24–26^. The HBC and HAC inhibitors in this study demonstrated higher efficiency (96.26% and 95.97%) compared to the previously listed organic inhibitors (91.8%, 93.2%, 92.8%, 94.3%, 92.5%, 93.7%, 94.1% and 94.3%). These findings suggest that this recent research serves as a logical progression of the study on organic inhibitors.

**Table 8:** Comparison of the inhibitory effectiveness obtained from EIS of several inhibitors used for different mild steel corrosion in various environments.

Inhibitor	Conc	Measurement method	Medium	Inhibition efficiency	References
Benzaldehyde thiosemicarbazone (BST)	300 × 10^–6^ M	EIS	0.5 M H_2_SO_4_	91.8	^24^
4-Carboxyl benzaldehyde thiosemicarbazone (PBST)	300 × 10^–6^ M	EIS	0.5 M H_2_SO_4_	93.2	^24^
2-Carboxyl benzaldehyde thiosemicarbazone (OCT)	300 × 10^–6^ M	EIS	0.5 M H_2_SO_4_	92.8	^24^
2-(2,4-Dimethoxybenzylidene)hydrazinecarbothioamide (DMBHC),	0.5 × 10^-3^M	EIS	1 N H_2_SO_4_	94.3	^25^
H-BT	400 × 10^–6^ M	EIS	1 M HCl	92.5	^26^
F-BT	400 × 10^–6^ M	EIS	1 M HCl	93.7	^26^
Cl-BT	400 × 10^–6^ M	EIS	1 M HCl	94.1	^26^
Br-BT	400 × 10^–6^ M	EIS	1 M HCl	94.3	^26^
HAC	800 ppm	EIS	0.5 M H_2_SO_4_	95.97	This work
HBC	800 ppm	EIS	0.5 M H_2_SO_4_	96.26	This work

## Conclusion

The prepared HAC and HBC compounds worked high effectively as inhibitors of MS corrosion in 1 M HCl, with the effectiveness of HAC and HBC inhibition efficiencies rising with increase in their amount in the corrosive solution. They classified as mixed-type, and Langmuir's isotherm is followed during adsorption. The study's negative ΔG_ads_ results show that spontaneous adsorption has occurred on the steel surface. The results of the DFT computation demonstrate that they had the ability to transfer electrons to the metal's surface. The MD and MC simulations show that the inhibitors have very flat surface adsorption geometries. This feature causes the adsorption centers of the inhibitor to be brought to the MS surface.

### Supplementary Information

Supplementary Information.

## Data Availability

The datasets generated during and/or analyzed during the current study are available from the corresponding author on reasonable request.

## References

[1] Alharthi NH, El-Hashemy MA, Derafa WM, Althobaiti IO, Altaleb HA (2022). Corrosion inhibition of mild steel by highly stable polydentate Schiff base derived from 1,3-propanediamine in aqueous acidic solution. J. Saudi Chem. Soc..

[2] Yousif QA (2023). Insight into the corrosion mitigation performance of three novel benzimidazole derivatives of amino acids for carbon steel (X56) in 1 M HCl solution. RSC Adv..

[3] Yousif QA, Bedair MA, Fadel Z, Al-Odail F, Abuelela AM (2024). Evaluating the efficacy of newly synthesized amino acid derivatives as corrosion inhibitors in acidic solutions. Inorg. Chem. Commun..

[4] El-Haddad MAM, Bahgat Radwan A, Sliem MH, Hassan WMI, Abdullah AM (2019). Highly efficient eco-friendly corrosion inhibitor for mild steel in 5 M HCl at elevated temperatures: Experimental & molecular dynamics study. Sci. Rep..

[5] Faiz M, Zahari A, Awang K, Hussin H (2020). Corrosion inhibition on mild steel in 1 M HCl solution by *Cryptocarya nigra* extracts and three of its constituents (alkaloids). RSC Adv..

[6] Hassan AMAM (2019). Synthesis of some triazole Schiff base derivatives and their metal complexes under Microwave irradiation and evaluation of their corrosion inhibition and biological activity. Egypt. J. Chem..

[7] Ayoola AA (2022). Corrosion inhibition of A36 mild steel in 0.5 M acid medium using waste citrus limonum peels. Results Eng..

[8] Sobhi M, Eid S (2018). Chemical, electrochemical and morphology studies on methyl hydroxyethyl cellulose as green inhibitor for corrosion of copper in hydrochloric acid solutions. Prot. Met. Phys. Chem. Surf..

[9] Bedair M (2015). Extracts of mint and tea as green corrosion inhibitors for mild steel in hydrochloric acid solution. Al-Azhar Bull. Sci..

[10] Elaryian HM, Bedair MA, Bedair AH, Aboushahba RM, Fouda AE-AS (2022). Synthesis, characterization of novel coumarin dyes as corrosion inhibitors for mild steel in acidic environment: Experimental, theoretical, and biological studies. J. Mol. Liq..

[11] Abdallah M, Kamar EM, Eid S, El-Etre AY (2016). Animal glue as green inhibitor for corrosion of aluminum and aluminum-silicon alloys in sodium hydroxide solutions. J. Mol. Liq..

[12] Seyam DFH, Tantawy A, Eid S, El-Etre AY (2022). Study of the inhibition effect of two novel synthesized amido-amine-based cationic surfactants on aluminum corrosion in 0.5 M HCl solution. J. Surf. Deterg..

[13] Silva ÁRL, Martínez-Huitle CA (2021). Theoretical studies of dimers and properties of the corrosion inhibitor profile for semicarbazones and thiosemicarbazones. J. Mol. Liq..

[14] Stanly Jacob K, Parameswaran G (2010). Corrosion inhibition of mild steel in hydrochloric acid solution by Schiff base furoin thiosemicarbazone. Corros. Sci..

[15] Khaled KFF (2010). Electrochemical behavior of nickel in nitric acid and its corrosion inhibition using some thiosemicarbazone derivatives. Electrochim. Acta.

[16] Parrilha GL, dos Santos RG, Beraldo H (2022). Applications of radiocomplexes with thiosemicarbazones and bis(thiosemicarbazones) in diagnostic and therapeutic nuclear medicine. Coord. Chem. Rev..

[17] Priyarega S, Haribabu J, Karvembu R (2022). Development of thiosemicarbazone-based transition metal complexes as homogeneous catalysts for various organic transformations. Inorgan. Chim. Acta.

[18] Bai C (2021). Synthesis and evaluation of novel thiosemicarbazone and semicarbazone analogs with both anti-proliferative and anti-metastatic activities against triple negative breast cancer. Bioorg. Med. Chem..

[19] Pandey V, Sharma K, Raghav N (2022). Ligand-based modeling of semicarbazones and thiosemicarbazones derivatives as Cathepsin B, H, and L inhibitors: A multi-target approach. J. Mol. Struct..

[20] Prajapati NP, Patel HD (2019). Novel thiosemicarbazone derivatives and their metal complexes: Recent development. Synth. Commun..

[21] Belicchi-Ferrari M, Bisceglie F, Pelosi G, Pinelli S, Tarasconi P (2007). Synthesis, characterization, crystal structure and antiproliferative in vitro activity of long-chain aliphatic thiosemicarbazones and their Ni(II) complexes. Polyhedron.

[22] Jia X (2020). Synthesis, cytotoxicity, and in vivo antitumor activity study of parthenolide semicarbazones and thiosemicarbazones. Bioorg. Med. Chem..

[23] Cheng W (2021). Design, synthesis and insecticidal activity of novel semicarbazones and thiosemicarbazones derived from chalcone. Nat. Prod. Res..

[24] Zhang, H. H., Qin, C. K., Chen, Y. & Zhang, Z. Inhibition behaviour of mild steel by three new benzaldehyde thiosemicarbazone derivatives in 0.5 M H_2_SO_4_ : Experimental and computational study. *R. Soc. Open Sci.* **6**, 190192 (2019). 10.1098/rsos.190192 PMC6731740 31598232

[25] Jawad, A.Q. *et al.* Synthesis, characterization, and corrosion inhibition potential of novel thiosemicarbazone on mild steel in sulfuric acid environment. *Coatings* **9**, 729 (2019).

[26] Zhang H (2022). Inhibition performance of halogen-substituted benzaldehyde thiosemicarbazones as corrosion inhibitors for mild steel in hydrochloric acid solution. RSC Adv..

[27] Dennington R, Keith TA, Millam JM (2016). GaussView 6.

[28] Frisch, M. & Clemente, F. *Gaussian 09, Revision A. 01* (Frisch, M.J., Trucks, G.W., Schlegel, H.B., Scuseria, G.E., Robb, M.A., Cheeseman, J.R., Scalmani, G. V., Barone, B., Mennucci, G.A., Petersson, H., Nakatsuji, M., Caricato, X., Li, H.P., Hratchian, A.F., Izmaylov, J., Bloino, G. eds.) (2009).

[29] Becke AD (1993). A new mixing of Hartree-Fock and local density-functional theories. J. Chem. Phys..

[30] Lee C, Yang W, Parr RG (1988). Development of the Colle-Salvetti correlation-energy formula into a functional of the electron density. Phys. Rev. B.

[31] Dassault Systems Materials Studio. (BIOVIA, 2017).

[32] Abbas MA (2022). Performance assessment by experimental and Theoretical approaches of newly synthetized benzyl amide derivatives as corrosion inhibitors for carbon steel in 1.0 M hydrochloric acid environment. Inorg. Chem. Commun..

[33] Anderson AA, Goetzen T, Shackelford SA, Tsank S (2000). A convenient one-step synthesis of 2-hydroxy-1,3,5-benzenetricarbaldehyde. Synth. Commun..

[34] Kudo E, Sasaki K, Kawamata S, Yamamoto K, Murahashi T (2021). Selective E to Z isomerization of 1,3-dienes enabled by a dinuclear mechanism. Nat. Commun..

[35] Oliveira P (2017). Mechanochemical synthesis and biological evaluation of novel isoniazid derivatives with potent antitubercular activity. Molecules.

[36] Nady H, Elgendy A, Arafa WAA, Gad ES (2022). Insight into the inhibition performance of thiosemicarbazones as efficient inhibitors for copper in acidic environment: Combined experimental and computational investigations. Colloids Surf. A Physicochem. Eng. Asp..

[37] Sherif E-SM (2006). Effects of 2-amino-5-(ethylthio)-1,3,4-thiadiazole on copper corrosion as a corrosion inhibitor in 3% NaCl solutions. Appl. Surf. Sci..

[38] Hu L, Zhang S, Li W, Hou B (2010). Electrochemical and thermodynamic investigation of diniconazole and triadimefon as corrosion inhibitors for copper in synthetic seawater. Corros. Sci..

[39] Bedair MA (2024). Highly effective inhibition of steel corrosion in 1.0 M HCl solution using a novel non-ionic surfactant with coumarin moiety: Practical and computational studies. Mater. Chem. Phys..

[40] Abuelela AM (2023). Electrochemical and DFT studies of *Terminalia bellerica* fruit extract as an eco-friendly inhibitor for the corrosion of steel. Sci. Rep..

[41] Ahmed AH, Sherif E-SM, Abdo HS, Gad ES (2021). Ethanedihydrazide as a corrosion inhibitor for iron in 35% NaCl solutions. ACS Omega.

[42] Yousif QA, Majeed MN, Bedair MA (2022). Surface protection against corrosion of Ni turbine blades by electrophoretic deposition of MnO_2_, TiO_2_ and TiO_2_–C nanocoating. RSC Adv..

[43] Li W, He Q, Zhang S, Pei C, Hou B (2008). Some new triazole derivatives as inhibitors for mild steel corrosion in acidic medium. J. Appl. Electrochem..

[44] Ashmawy AM, Said R, Naguib IA, Yao B, Bedair MA (2022). Anticorrosion study for brass alloys in heat exchangers during acid cleaning using novel gemini surfactants based on benzalkonium tetrafluoroborate. ACS Omega.

[45] Yan Y, Li W, Cai L, Hou B (2008). Electrochemical and quantum chemical study of purines as corrosion inhibitors for mild steel in 1M HCl solution. Electrochim. Acta.

[46] Li Y, Zhao P, Liang Q, Hou B (2005). Berberine as a natural source inhibitor for mild steel in 1M H2SO4. Appl. Surf. Sci..

[47] Mansfeld F, Shih H (1988). Detection of pitting with electrochemical impedance spectroscopy. J. Electrochem. Soc..

[48] Badawy WA, El-Rabiei MM, Nady H (2014). Synergistic effects of alloying elements in Cu-ternary alloys in chloride solutions. Electrochim. Acta.

[49] El-Hafez GMA, Badawy WA (2013). The use of cysteine, N-acetyl cysteine and methionine as environmentally friendly corrosion inhibitors for Cu–10Al–5Ni alloy in neutral chloride solutions. Electrochim. Acta.

[50] Majeed MN, Yousif QA, Bedair MA (2022). Study of the corrosion of nickel-chromium alloy in an acidic solution protected by nickel nanoparticles. ACS Omega.

[51] Wang J (2022). Insight into the origin of pseudo peaks decoded by the distribution of relaxation times/ differential capacity method for electrochemical impedance spectroscopy. J. Electroanal. Chem..

[52] Hegde M, Nayak SP (2019). Aqueous extract of dillenia pentagyna fruit as green inhibitor for mild steel corrosion in 0.5 m hydrochloric acid solution. J. Mater. Environ. Sci..

[53] Sherif EM, Park S-M (2006). Inhibition of copper corrosion in acidic pickling solutions by *N*-phenyl-1,4-phenylenediamine. Electrochim. Acta.

[54] Bedair MA, Soliman SA, Hegazy MA, Obot IB, Ahmed AS (2019). Empirical and theoretical investigations on the corrosion inhibition characteristics of mild steel by three new Schiff base derivatives. J. Adhes. Sci. Technol..

[55] Yadav AP, Nishikata A, Tsuru T (2004). Electrochemical impedance study on galvanized steel corrosion under cyclic wet–dry conditions––Influence of time of wetness. Corros. Sci..

[56] Bedair, M. A., Soliman, S. A. & Metwally, M. S. Synthesis and characterization of some nonionic surfactants as corrosion inhibitors for steel in 1.0 M HCl (Experimental and computational study). *J. Ind. Eng. Chem.* **41**, 10–22 (2016).

[57] Cen H, Zhang X, Zhao L, Chen Z, Guo X (2019). Carbon dots as effective corrosion inhibitor for 5052 aluminium alloy in 0.1 M HCl solution. Corros. Sci..

[58] Ragab M, Bedair MA (2023). The effect of permanent magnet stirring on the corrosion resistance of Sn-2.0Ag-0.5Cu-2Zn solder alloys in NaCl solution. Mater. Chem. Phys..

[59] Badr EA, Bedair MA, Shaban SM (2018). Adsorption and performance assessment of some imine derivatives as mild steel corrosion inhibitors in 1.0 M HCl solution by chemical, electrochemical and computational methods. Mater. Chem. Phys..

[60] Ye Y (2020). A high-efficiency corrosion inhibitor of N-doped citric acid-based carbon dots for mild steel in hydrochloric acid environment. J. Hazard. Mater..

[61] Soliman SAA, Metwally MSS, Selim SRR, Bedair MAA, Abbas MA (2014). Corrosion inhibition and adsorption behavior of new Schiff base surfactant on steel in acidic environment: Experimental and theoretical studies. J. Ind. Eng. Chem..

[62] Mostafa MAA, Ashmawy AM, Reheim MAMMA, Bedair MA, Abuelela AM (2021). Molecular structure aspects and molecular reactivity of some triazole derivatives for corrosion inhibition of aluminum in 1 M HCl solution. J. Mol. Struct..

[63] Awad, M. K. M. K., Metwally, M. S. S. M., Soliman, S. A. S. A., El-Zomrawy, A. A. A. A. A. & bedair, M. A. M. A. Experimental and quantum chemical studies of the effect of poly ethylene glycol as corrosion inhibitors of aluminum surface. *J. Ind. Eng. Chem.* **20**, 796–808 (2014).

[64] Hamani H (2014). Electrochemical and quantum chemical studies of some azomethine compounds as corrosion inhibitors for mild steel in 1M hydrochloric acid. Corros. Sci..

[65] Bedair MA (2016). The effect of structure parameters on the corrosion inhibition effect of some heterocyclic nitrogen organic compounds. J. Mol. Liq..

[66] Bedair MA, Abuelela AM, Alshareef M, Owda M, Eliwa EM (2023). Ethyl ester/acyl hydrazide-based aromatic sulfonamides: facile synthesis, structural characterization, electrochemical measurements and theoretical studies as effective corrosion inhibitors for mild steel in 1.0 M HCl. RSC Adv..

[67] Melhi S (2022). Effective corrosion inhibition of mild steel in hydrochloric acid by newly synthesized Schiff base nano Co(ii) and Cr(iii) complexes: Spectral, thermal, electrochemical and DFT (FMO, NBO) studies. RSC Adv..

[68] Abbas MA (2022). Synthesis, characterization, thermodynamic analysis and quantum chemical approach of branched *N, N*′-bis(*p*-hydroxybenzoyl)-based propanediamine and triethylenetetramine for carbon steel corrosion inhibition in hydrochloric acid medium. Arab. J. Sci. Eng..

[69] Bedair MA (2023). Insights into the adsorption and corrosion inhibition properties of newly synthesized diazinyl derivatives for mild steel in hydrochloric acid: Synthesis, electrochemical, SRB biological resistivity and quantum chemical calculations. RSC Adv..

[70] Elaryian HM, Bedair MA, Bedair AH, Aboushahba RM, Fouda AE-AS (2022). Corrosion mitigation for steel in acid environment using novel *p*-phenylenediamine and benzidine coumarin derivatives: Synthesis, electrochemical, computational and SRB biological resistivity. RSC Adv..

[71] Bedair MA, Elaryian HM, Bedair AH, Aboushahba RM, El-Aziz S, Fouda A (2023). Novel coumarin-buta-1,3-diene conjugated donor–acceptor systems as corrosion inhibitors for mild steel in 1.0 M HCl: Synthesis, electrochemical, computational and SRB biological resistivity. Inorg. Chem. Commun..

[72] Alarfaji SS, Ali IH, Bani-Fwaz MZ, Bedair MA (2021). Synthesis and assessment of two malonyl dihydrazide derivatives as corrosion inhibitors for carbon steel in acidic media: Experimental and theoretical studies. Molecules.

[73] Zakaria K, Abbas MA, Bedair MA (2022). Herbal expired drug bearing glycosides and polysaccharides moieties as green and cost-effective oilfield corrosion inhibitor: Electrochemical and computational studies. J. Mol. Liq..

[74] Verma, D. K. Density functional theory (DFT) as a powerful tool for designing corrosion inhibitors in aqueous phase. In *Advanced Engineering Testing*10.5772/intechopen.78333 (InTech, 2018).

[75] Xu Z, Tan B, Zhang S, Chen J, Li W (2023). Exploring of an ecological corrosion inhibitor of wood hibiscus leaf extract for the Cu/H_2_SO_4_ system based on experimental study and theoretical calculations. J. Taiwan Inst. Chem. Eng..

[76] Bedair MA, Alosaimi EH, Melhi S (2021). A study of the inhibitive effect for corrosion of steel in 1.0 M HCl using a new nonionic surfactant based on coumarin moiety: Chemical, electrochemical and quantum mechanics calculations. J. Adhes. Sci. Technol..

[77] Bedair MA, Abuelela AM, Zoghaib WM, Mohamed TA (2021). Molecular structure, tautomer’s, reactivity and inhibition studies on 6-methyl-2-thiouracil for mild steel corrosion in aqueous HCl (1.00 M): Experimental and Theoretical Studies. J. Mol. Struct..

[78] Abbas MA, Bedair MA, El-Azabawy OE, Gad ES (2021). Anticorrosion effect of ethoxylate sulfanilamide compounds on carbon steel in 1 M hydrochloric acid: Electrochemical and theoretical studies. ACS Omega.

[79] Ramezanzadeh M, Bahlakeh G, Sanaei Z, Ramezanzadeh B (2019). Corrosion inhibition of mild steel in 1 M HCl solution by ethanolic extract of eco-friendly *Mangifera indica* (mango) leaves: Electrochemical, molecular dynamics, Monte Carlo and ab initio study. Appl. Surf. Sci..

[80] Saha SK, Dutta A, Ghosh P, Sukul D, Banerjee P (2016). Novel Schiff-base molecules as efficient corrosion inhibitors for mild steel surface in 1 M HCl medium: Experimental and theoretical approach. Phys. Chem. Chem. Phys..

[81] Gebril MA, Bedair MA, Soliman SA, Bakr MF, Mohamed MBI (2022). Experimental and computational studies of the influence of non-ionic surfactants with coumarin moiety as corrosion inhibitors for carbon steel in 1.0 M HCl. J. Mol. Liq..

[82] El Faydy M (2021). Insight into the corrosion inhibition of new bis-quinolin-8-ols derivatives as highly efficient inhibitors for C35E steel in 0.5 M H_2_SO_4_. J. Mol. Liq..

[83] Vranda Shenoy K, Venugopal PP, Reena Kumari PD, Chakraborty D (2021). Effective inhibition of mild steel corrosion by 6-bromo-(2,4-dimethoxyphenyl)methylidene]imidazo [1,2-a]pyridine-2-carbohydrazide in 0.5 M HCl: Insights from experimental and computational study. J. Mol. Struct..

[84] Abbas MA, Bedair MA (2019). Adsorption and computational studies for evaluating the behavior of silicon based compounds as novel corrosion inhibitors of carbon steel surfaces in acidic media. Z. Phys. Chem..

[85] Abdelsalam MM (2022). Green synthesis, electrochemical, and DFT studies on the corrosion inhibition of steel by some novel triazole Schiff base derivatives in hydrochloric acid solution. Arab. J. Chem..

[86] Gad ES, Abbas MA, Bedair MA, El-Azabawy OE, Mukhtar SM (2023). Synthesis and applications of novel Schiff base derivatives as corrosion inhibitors and additives for improvement of reinforced concrete. Sci. Rep..

[87] Abuelela AM, Bedair MA, Zoghaib WM, Wilson LD, Mohamed TA (2021). Molecular structure and mild steel/HCl corrosion inhibition of 4,5-Dicyanoimidazole: Vibrational, electrochemical and quantum mechanical calculations. J. Mol. Struct..

[88] Berdimurodov E (2022). Novel glycoluril pharmaceutically active compound as a green corrosion inhibitor for the oil and gas industry. J. Electroanal. Chem..

[89] Daoudi W (2023). Synthesis, characterization, and corrosion inhibition activity of new imidazo[1.2-a]pyridine chalcones. Mater. Sci. Eng. B.

[90] Bereket G, Yurt A (2001). The inhibition effect of amino acids and hydroxy carboxylic acids on pitting corrosion of aluminum alloy 7075. Corros. Sci..

