# Supplementary material for: Exploring the synthesis, characterization, and corrosion inhibition of new tris-thiosemicarbazone derivatives for acidic steel settings using computational and experimental studies
Source: Sci Rep. 2024 Jun 10;14:13310. doi: 10.1038/s41598-024-64199-x (PMC11164706; doi:10.1038/s41598-024-64199-x)
Supplement: Supplementary file 1 — Supplementary Information. [file 41598_2024_64199_MOESM1_ESM.docx]

**Supporting Information for**

**Exploring the synthesis, characterization, and corrosion inhibition of new tris-thiosemicarbazone derivatives for acidic steel settings using computational and experimental studies.**

**Ahmed M. Abuelela^a,*^, Mahmoud A. Bedair^b,*^, Ehab S. Gad^c,*^,** **Y. F. El-Aryan^b^,** **Wael Abdelgayed Ahmed Arafa****^[d](https://www.sciencedirect.com/science/article/pii/S1319610320301484#!),c^, Asmaa K. Mourad^d^,** **H. Nady^c,d^, Salah Eid^c,e^**

^a^Department of Chemistry, College of Science, King Faisal University, Al-Hassa, 31982, Saudi Arabia.

^b^Department of Chemistry, College of Science, University of Bisha, P.O. Box 511, Bisha, 61922, Saudi Arabia.

^c^Chemistry Department, College of Science, Jouf University, P. O. Box 2014, Sakaka, Jouf, Saudi Arabia.

^d^Chemistry Department, Faculty of Science, Fayoum University, P. O. Box 63514, Fayoum, Egypt.

^e^Chemistry Department, Faculty of science, Benha University, Benha, Egypt.

**Table S1** Calculated NBOs densities of *HAC* at expected inhibitor-metal interactions.

| LP (1) C_1_ | LP (2) S_24_ |
| --- | --- |
| 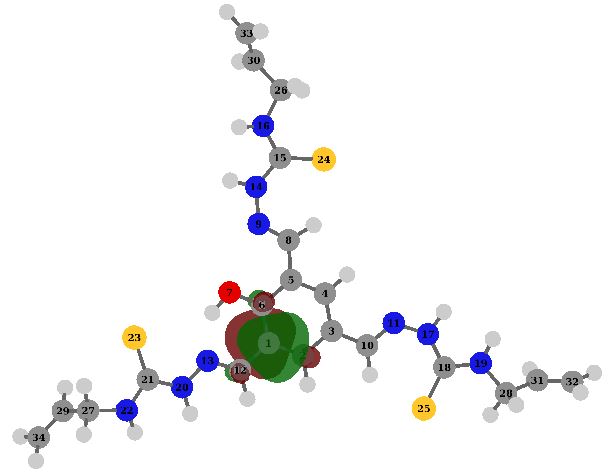 | 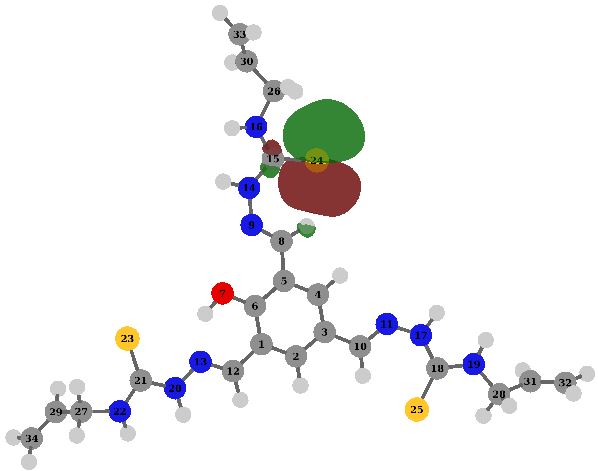 |
| LP (2) S_23_ | LP (2) S_25_ |
| 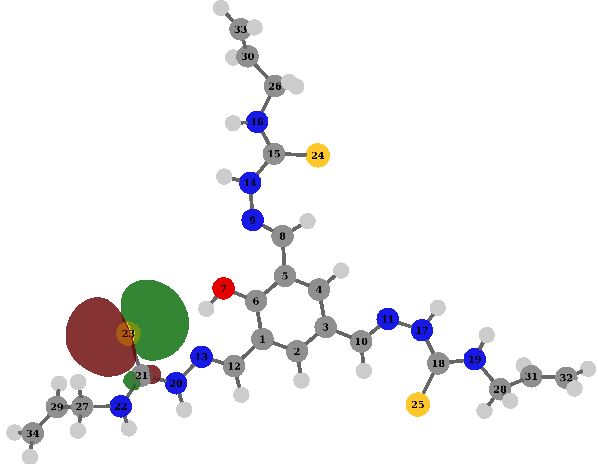 | 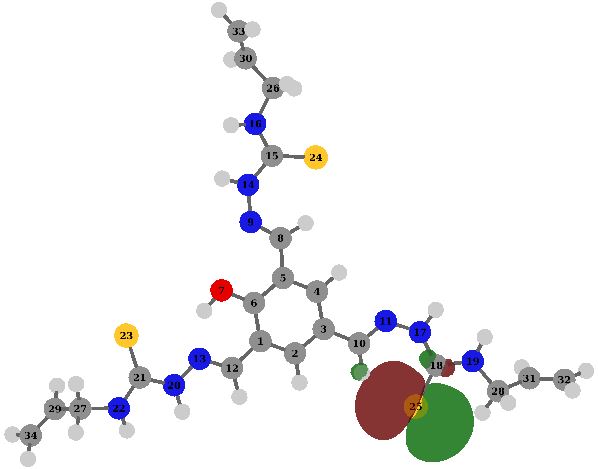 |
| BD (2) C_4_-C_5_ | BD (2) C_2_-C_3_ |
| 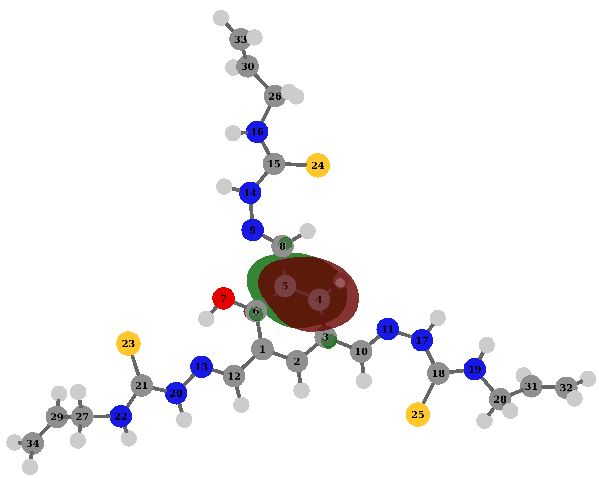 | 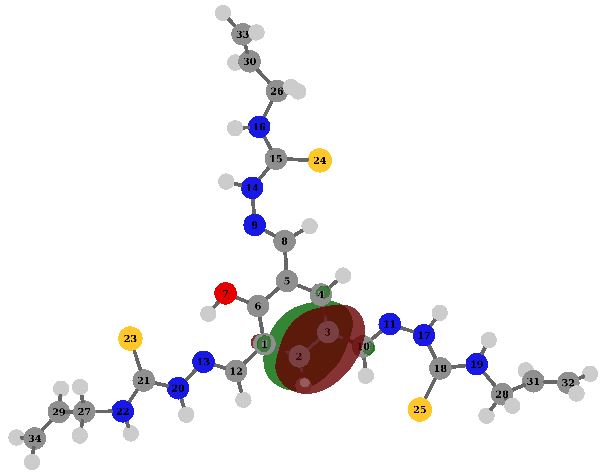 |
| LP (1) N_16_ | LP (1) N_14_ |
| 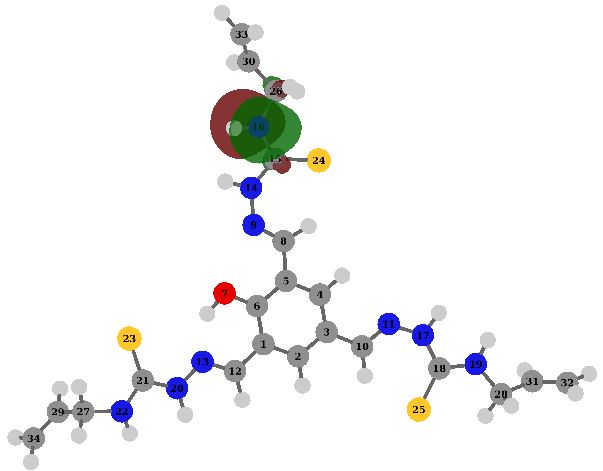 | 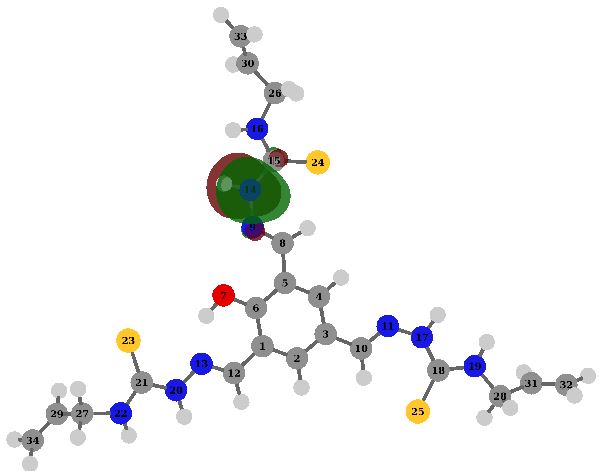 |
| LP (1) N_19_ | LP (1) N_17_ |
| 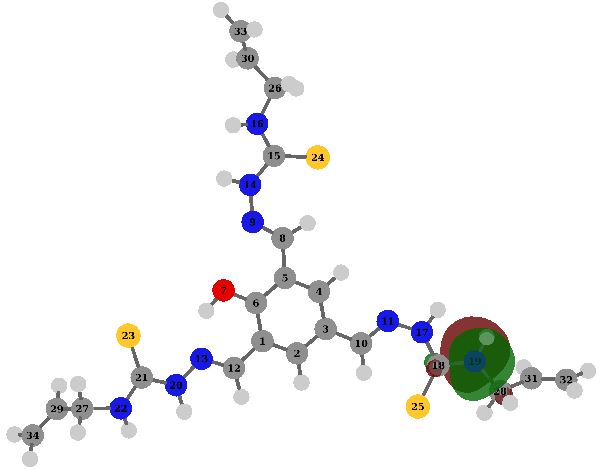 | 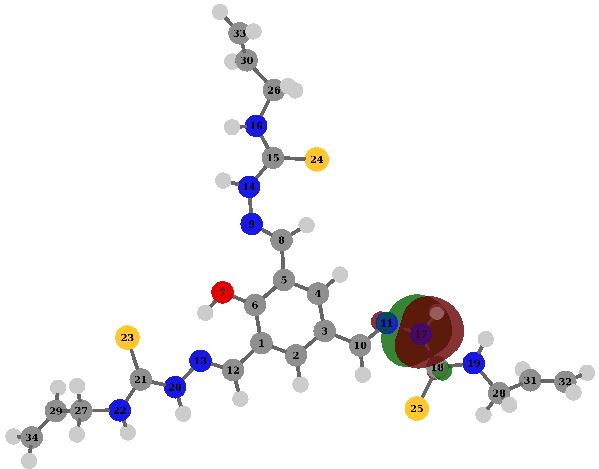 |
| LP (1) N_22_ | BD (2) C_30_-C_33_ |
| 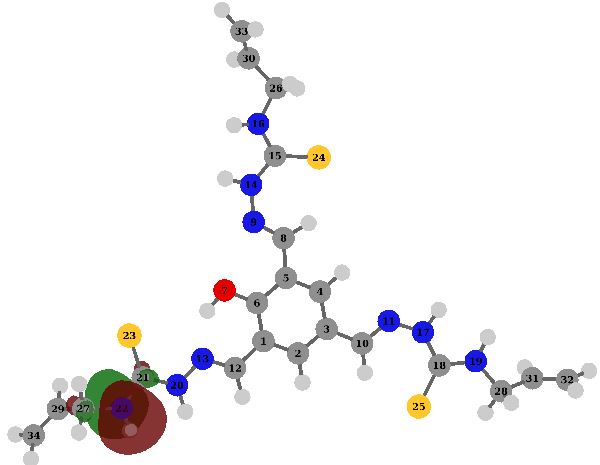 | 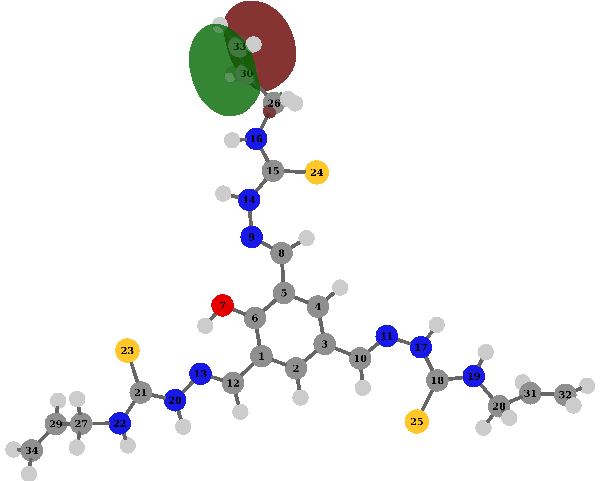 |
| BD (1) C_21_-S_23_ | BD (2) C_31_-C_32_ |
| 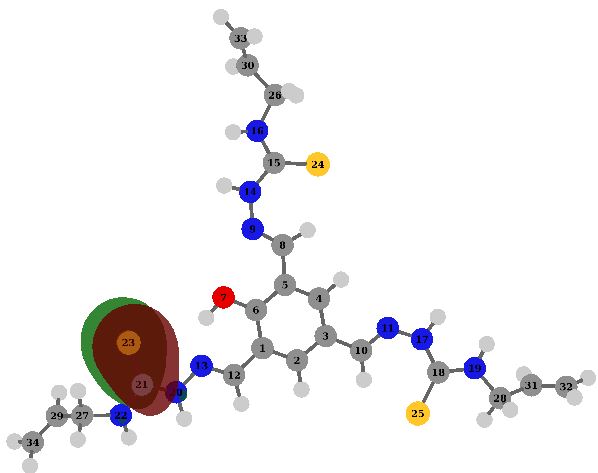 | 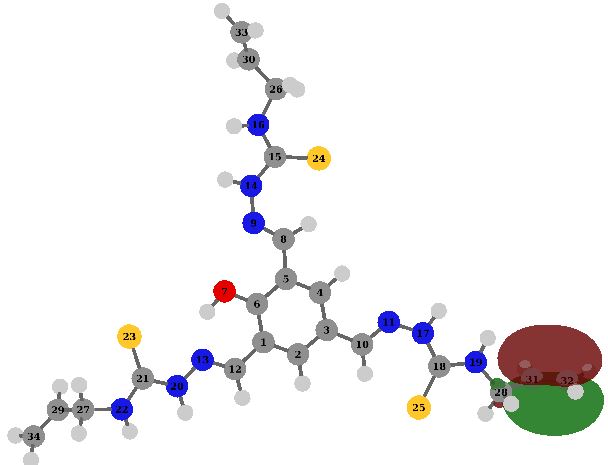 |
| BD (2) C_29_-C_34_ | LP (1) N_20_ |
| 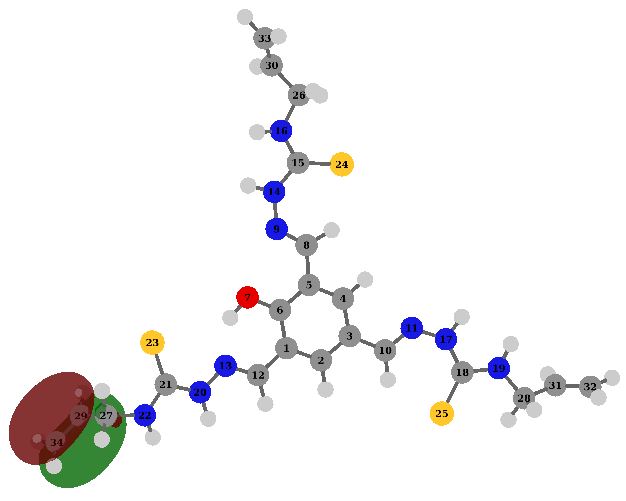 | 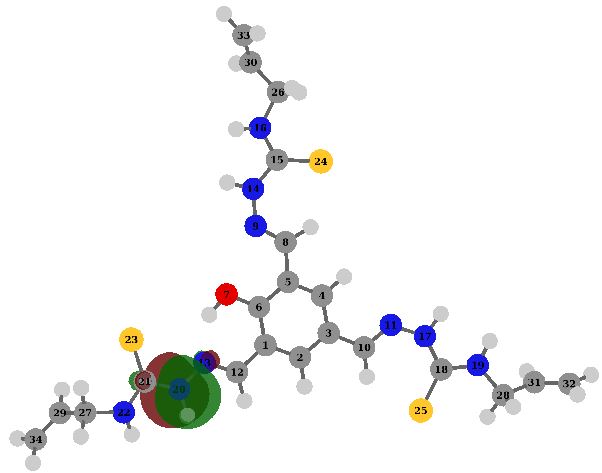 |

**Table S2** Calculated NBOs densities of *p*-TSAE at expected inhibitor-metal interactions.

| LP (1) C_1_ | LP (2) S_25_ |
| --- | --- |
| 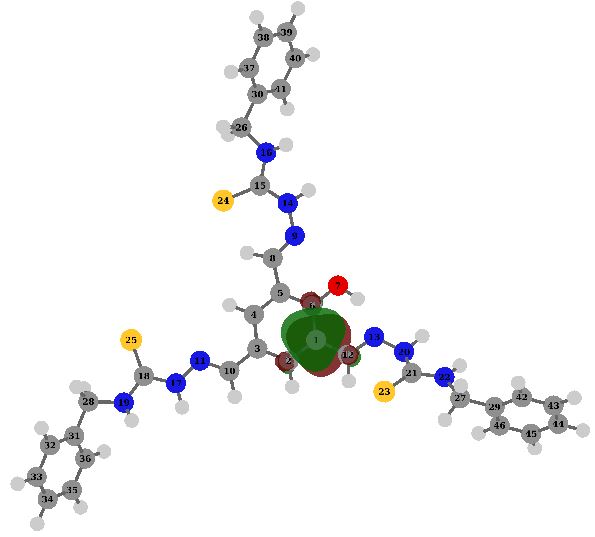 | 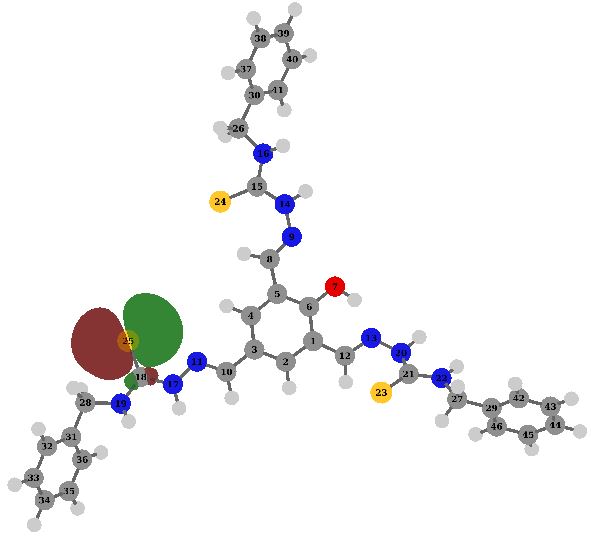 |
| LP (2) S_24_ | LP (2) S_23_ |
| 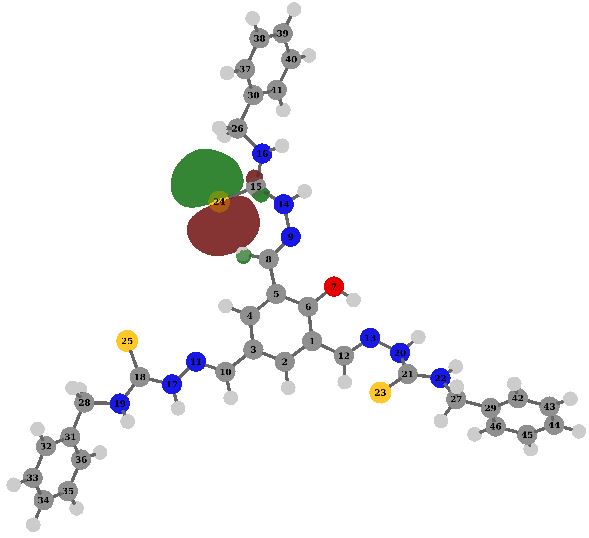 | 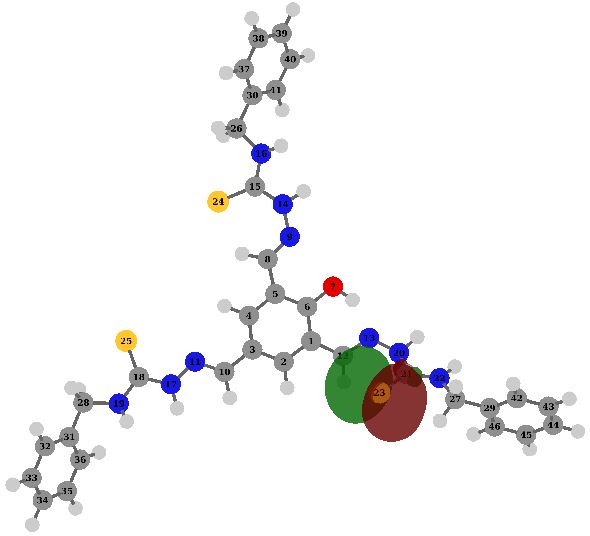 |
| BD (2) C_4_-C_5_ | BD (2) C_33_-C_34_ |
| 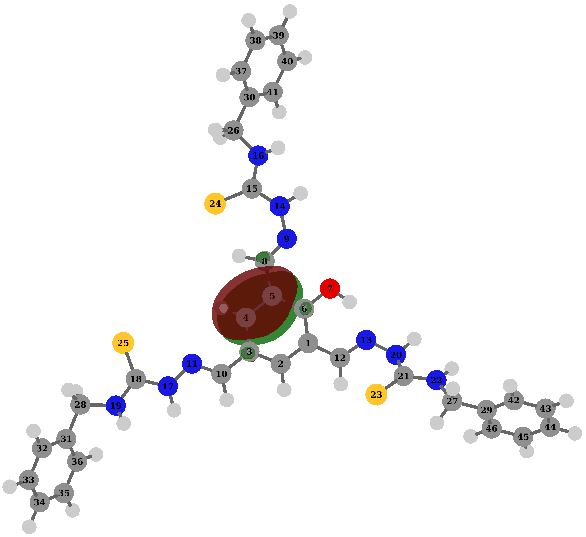 | 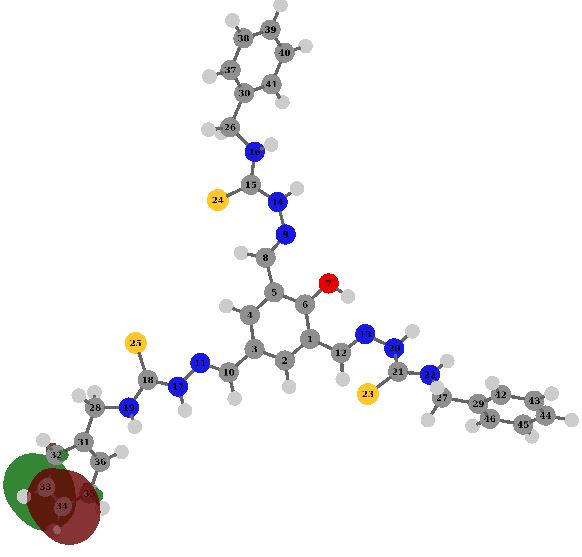 |
| BD (2) C_38_-C_39_ | BD (2) C_31_-C_32_ |
| 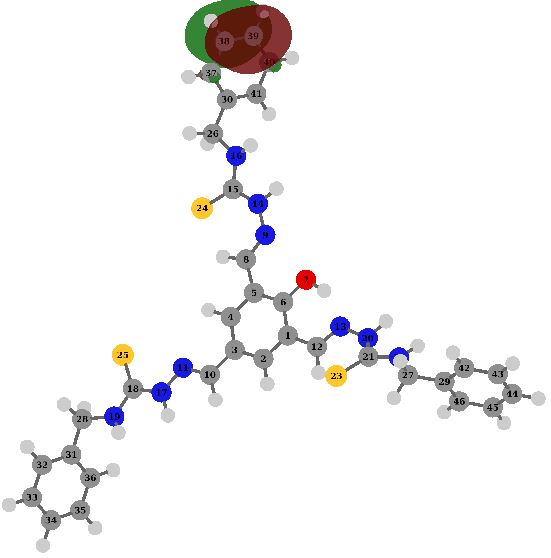 | 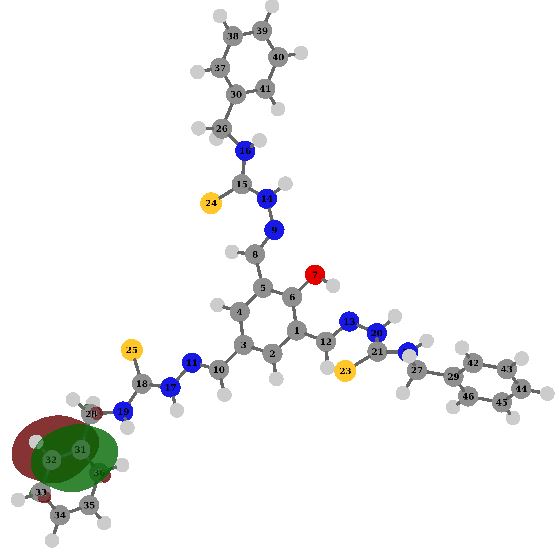 |
| BD (2) C_35_-C_36_ | BD (2) C_30_-C_37_ |
| 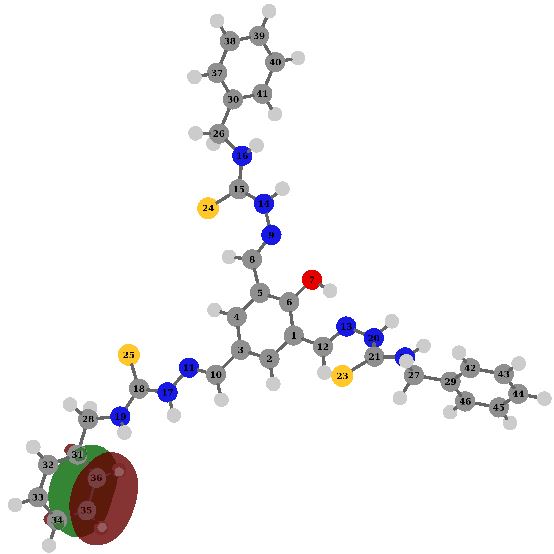 | 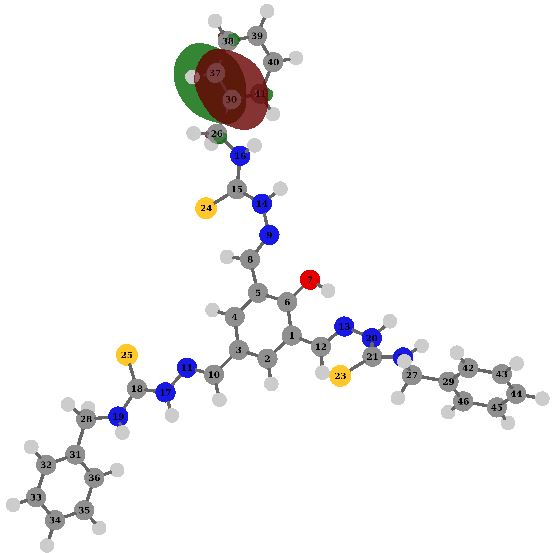 |
| BD (2) C_40_-C_41_ | BD (2) C_2_-C_3_ |
| 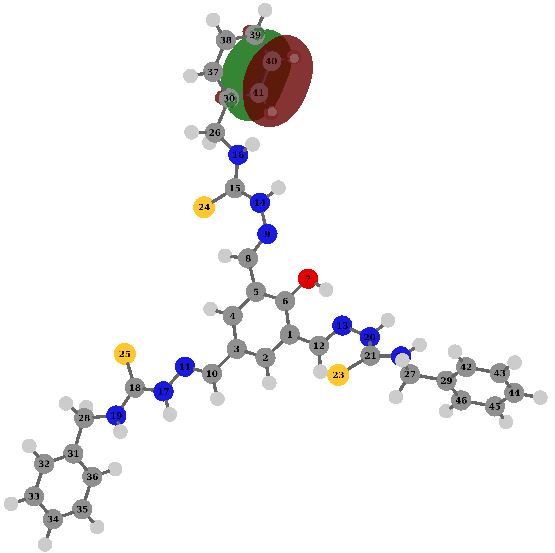 | 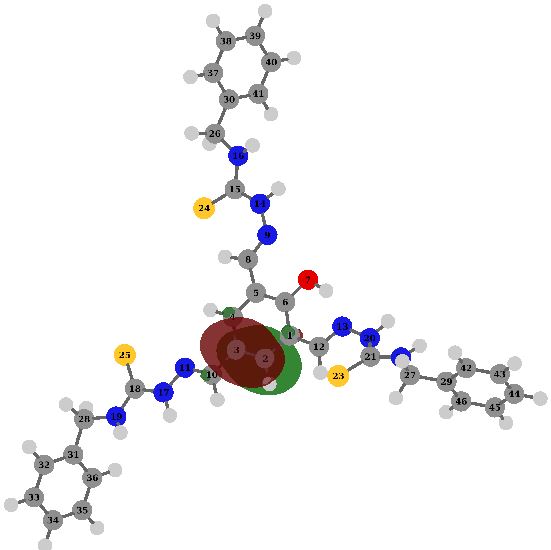 |
| BD (2) C_43_-C_44_ | BD (2) C_45_-C_46_ |
| 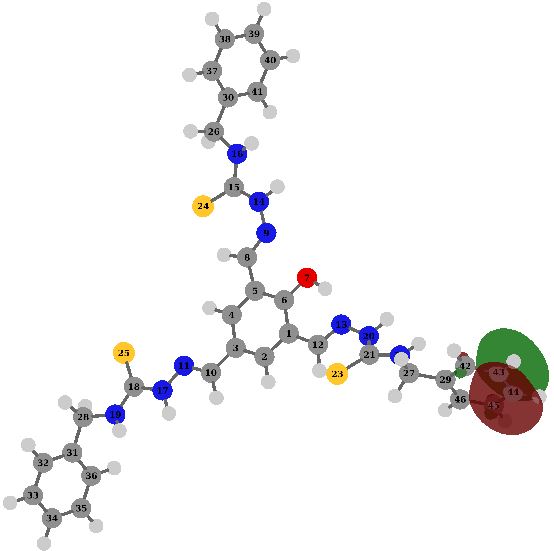 | 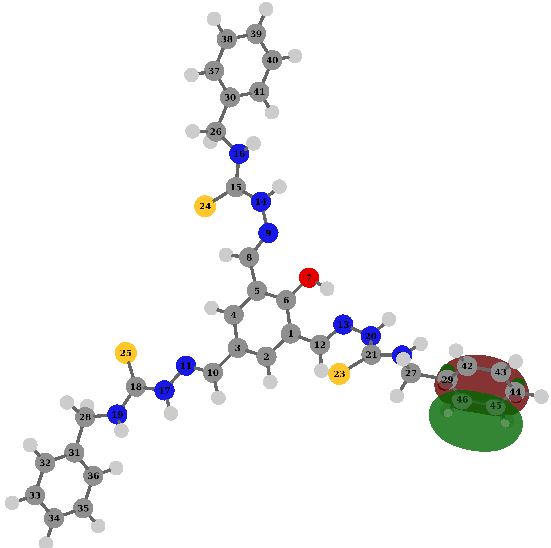 |
| BD (2) C_29_-C_42_ | LP (1) N_14_ |
| 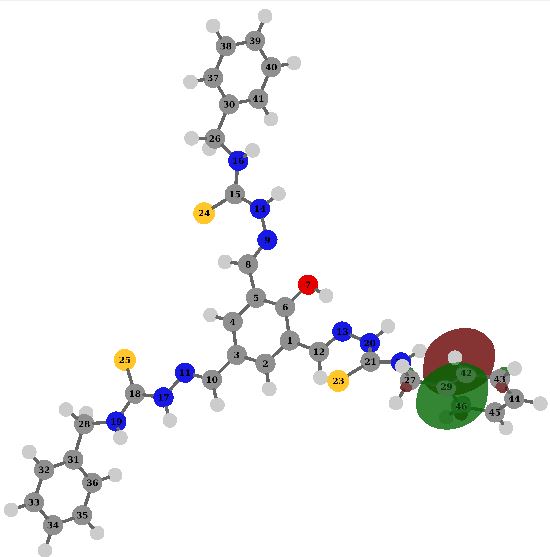 | 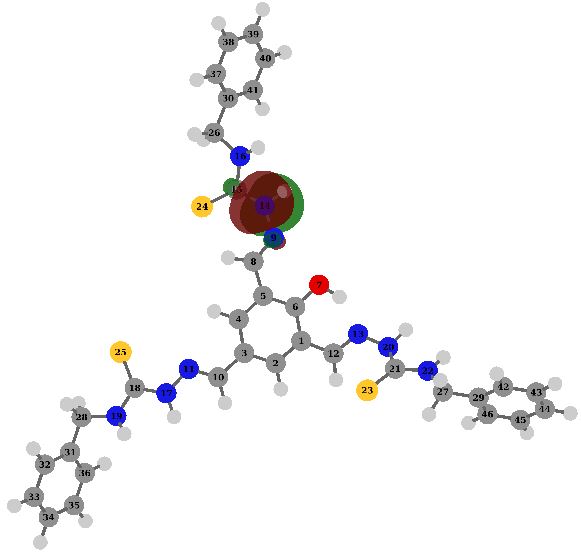 |


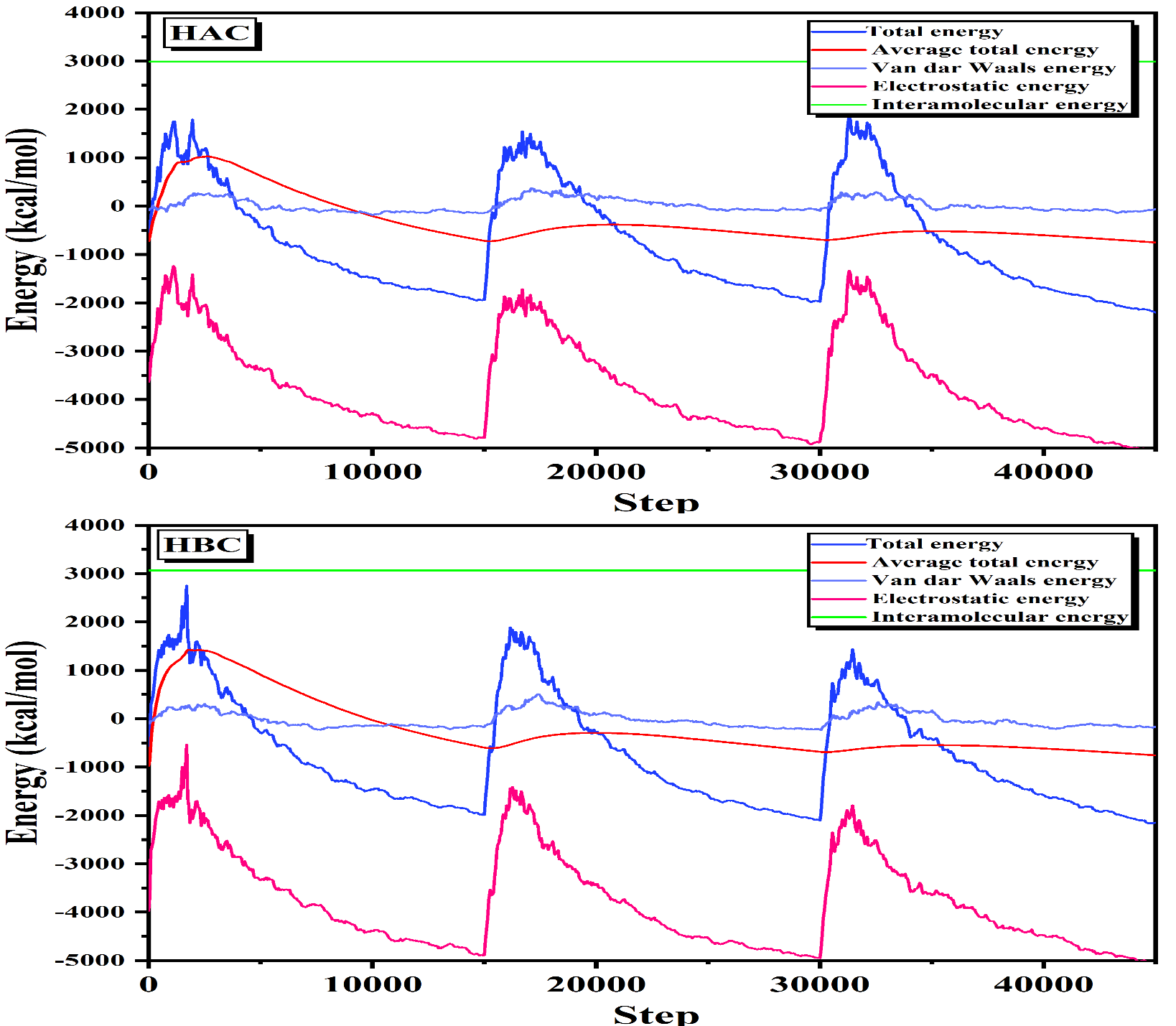
**Figure S1.**  Distribution of the different energy terms during the process of optimization of the adsorption of for (HAC-H^+^ and HBC-H^+^/ 200 H_2_O/ 19 H3O^+^/ 10 SO_4_^--^) systems onto the Fe (110) surface obtained by via MC.


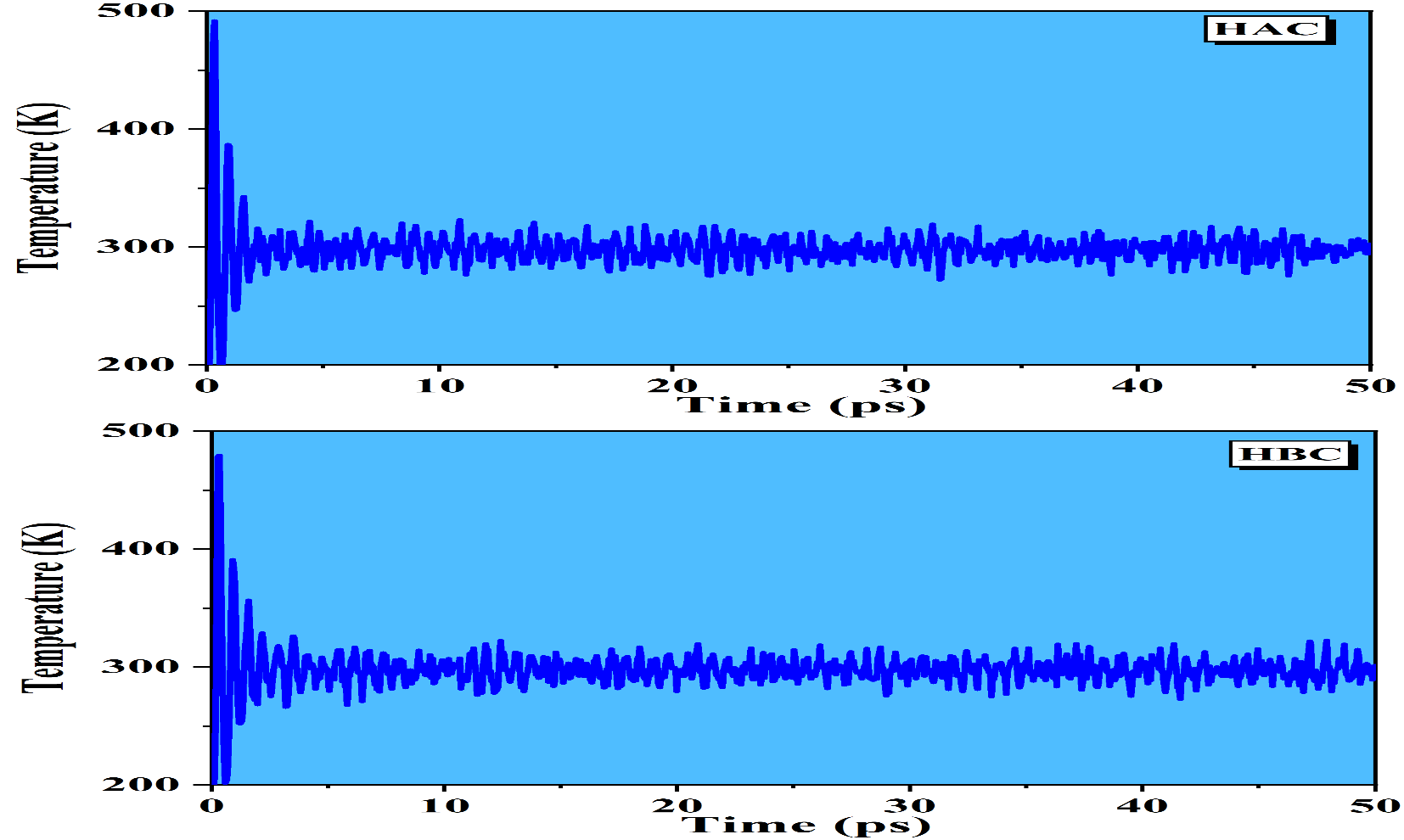
**Figure S2.**  Temperature fluctuation at T=298 K for (HAC-H^+^ and HBC-H^+^/ 200 H_2_O/ 19 H3O^+^/ 10 SO_4_^--^) systems onto the Fe (110) surface, obtained via MD.
